# Boundary cap cells constrain spinal motor neuron somal migration at motor exit points by a semaphorin-plexin mechanism

**DOI:** 10.1186/1749-8104-2-21

**Published:** 2007-10-30

**Authors:** Romke Bron, Matthieu Vermeren, Natalie Kokot, William Andrews, Graham E Little, Kevin J Mitchell, James Cohen

**Affiliations:** 1 MRC Centre for Developmental Neurobiology, King's College London, Guy's Campus, London Bridge, London, SE1 1UL, UK; 2 Department of Physiology, Development and Neurosciences, University of Cambridge, Downing Street, Cambridge, CB2 3DY, UK; 3 Department of Anatomy and Developmental Biology, University College London, London, WC1E 6BT, UK; 4 Smurfit Institute of Genetics and Institute of Neuroscience, Trinity College Dublin, Dublin 2, Ireland; 5 Department of Anatomy and Cell Biology, University of Melbourne, Parkville, VIC 3010, Australia

## Abstract

**Background:**

In developing neurons, somal migration and initiation of axon outgrowth often occur simultaneously and are regulated in part by similar classes of molecules. When neurons reach their final destinations, however, somal translocation and axon extension are uncoupled. Insights into the mechanisms underlying this process of disengagement came from our study of the behaviour of embryonic spinal motor neurons following ablation of boundary cap cells. These are neural crest derivatives that transiently reside at motor exit points, central nervous system (CNS):peripheral nervous system (PNS) interfaces where motor axons leave the CNS. In the absence of boundary cap cells, motor neuron cell bodies migrate along their axons into the periphery, suggesting that repellent signals from boundary cap cells regulate the selective gating of somal migration and axon outgrowth at the motor exit point. Here we used RNA interference in the chick embryo together with analysis of null mutant mice to identify possible boundary cap cell ligands, their receptors on motor neurons and cytoplasmic signalling molecules that control this process.

**Results:**

We demonstrate that targeted knock down in motor neurons of Neuropilin-2 (Npn-2), a high affinity receptor for class 3 semaphorins, causes their somata to migrate to ectopic positions in ventral nerve roots. This finding was corroborated in *Npn-2 *null mice, in which we identified motor neuron cell bodies in ectopic positions in the PNS. Our RNA interference studies further revealed a role for Plexin-A2, but not Plexin-A1 or Plexin-A4. We show that chick and mouse boundary cap cells express Sema3B and 3G, secreted semaphorins, and Sema6A, a transmembrane semaphorin. However, no increased numbers of ectopic motor neurons are found in *Sema3B *null mouse embryos. In contrast, *Sema6A *null mice display an ectopic motor neuron phenotype. Finally, knockdown of MICAL3, a downstream semaphorin/Plexin-A signalling molecule, in chick motor neurons led to their ectopic positioning in the PNS.

**Conclusion:**

We conclude that semaphorin-mediated repellent interactions between boundary cap cells and immature spinal motor neurons regulates somal positioning by countering the drag exerted on motor neuron cell bodies by their axons as they emerge from the CNS at motor exit points. Our data support a model in which BC cell semaphorins signal through Npn-2 and/or Plexin-A2 receptors on motor neurons via a cytoplasmic effector, MICAL3, to trigger cytoskeletal reorganisation. This leads to the disengagement of somal migration from axon extension and the confinement of motor neuron cell bodies to the spinal cord.

## Background

The migration of neurons is a key process in the development of the nervous system since sites of neurogenesis are often separated by long distances from final destinations. Neuron migration is complex, requiring synchronisation of multiple stepwise processes that differ in important respects from other types of migrating cells. In non-neuronal cells, nucleokinesis or somal translocation is tightly coupled to that of the exploratory lamellopodia. Neuron migration, in contrast, is initiated independently of the cell soma by the extension of long processes preceded by an exploratory growth cone [1]. Somal translocation occurs only after the leading process becomes consolidated by sustained movement in one direction [2-4]. On reaching its destination, the cell body stops and somal migration and axonal extension become irreversibly disengaged by unknown mechanisms. Since the guidance of migrating neurons appears to be regulated by cues similar to those that govern the guidance of axons, how do these mechanisms become uncoupled within individual post-migratory neurons? For example, motor neuron subtypes arise from a common source of ventricular zone progenitors that migrate into the ventral neural tube where they are initially intermingled. Later, however, motor neurons with similar muscle targets and sensory afferent inputs cluster together into columns and sort out into distinct pools [5]. Expression of a combinatorial code of transcription factors belonging to the ETS and homeodomain families regulates both the distinct settling positions of motor neuron soma within the ventral spinal cord and the targeting of their axons in the periphery [6]. Evidence suggests that this may be achieved by the column- and pool-specific expression of receptors for guidance cues that may be distinct from those that control axon guidance. Thus, EphrinA- EphA4 interactions regulate axon guidance along the dorsoventral axis of limbs but appear to have no influence on motor neuron settling positions [7]. Similarly, the chemokine receptor Cxcr4 has been implicated in the choice between dorsal and ventral exit points by the axons of spinal accessory motor neurons but does not affect migration of their soma [8,9]. Conversely, class II cadherins play an important part in pool-specific sorting of motor neuron subtypes but have not so far been implicated in the control of motor axon guidance [10]. In other instances, however, both axon guidance and cell positioning mechanisms rely on the same ligand-receptor complexes. Thus, in zebrafish neuropilin (Npn)-1a morphants, motor axons have abnormal branching and exit the spinal cord at inappropriate levels whilst at the same time the somata of some motor neurons migrate to ectopic positions [11]. Likewise, in *Caenorhabditis elegans*, mutation in the Eph kinase homologue vab-1 causes both the anterior overshooting of mechanosensor posterior lateral motor (PLM) axons and the mispositioning of PLM neurons [12].

The question of the uncoupling of axonal growth and cell body stabilisation in post-migratory neurons is highlighted in the case of developing spinal motor neurons whose cell bodies and axons come to reside in separate nervous system compartments, that is, the central nervous system (CNS) and peripheral nervous system (PNS) respectively. We previously described evidence that after motor axons first emerge from the CNS but before motor neurons acquire their final settling positions, interactions with neural crest derivatives located at motor exit points, boundary cap (BC) cells, influence somal migration. Ablation of BC cells led to the ectopic positioning of many motor neuron cell bodies that escape the spinal cord by apparently translocating along their axons into ventral nerve roots. This result suggested that disruption of this interaction influenced a key process in the maturation of motor neurons: the positional stabilisation of their soma. We proposed that the mechanism for uncoupling somal migration from axonal extension in immature motor neurons requires localised repellent signalling from BC cells to restrain the innate tendency of cell bodies to follow the path taken by their axons. In the search for possible repellents that could mediate this process, several lines of evidence suggest the semaphorin family and its receptors deserve closer scrutiny. Thus, semaphorins and their multimeric surface receptors, neuropilins and/or plexins, have been implicated in the control of the guidance, fasciculation and target selection of motor axons in the periphery [13] and have been suggested to be involved in motor pool sorting [14,15]. Also, both secreted and membrane bound semaphorins have been implicated more generally in neuronal migration, including the sorting of striatal from cortical interneurons [16] and the radial migration of cerebellar granule cells [17].

Here we use genetic approaches in chick and mouse embryos to show that BC cell-motor neuron interactions that regulate somal migration are mediated by neuropilin and/or plexin interactions with semaphorin ligands. We show that targeted knock down in motor neurons of Npn-2, a high affinity co-receptor for secreted class 3 semaphorins, cause motor neuron cell bodies to migrate to ectopic positions in ventral nerve roots, a finding corroborated in *Npn-2 *null mice. BC cells express semaphorin-3B, a Npn-2 ligand, and semaphorin 6A, a transmembrane semaphorin. Ectopic motor neurons are found when *Sema6A *short hairpin RNA (shRNA) but not *Sema3B *shRNA is expressed in chick neural crest and in *Sema6A *but not *Sema3B *null mice. RNA interference (RNAi) in chick targeting potential Npn-2 and Sema6A co-receptors reveals a role for Plexin-A2 but not Plexin-A1 or Plexin-A4. The knockdown of MICAL3, a cytoplasmic semaphorin/plexin signalling molecule, in motor neurons results in a very large number of ectopic motor neuron somata in the PNS. Together, our data point to the involvement of BC cell semaphorin-induced signalling pathways controlling motor neuron somal positioning in the embryonic ventral spinal cord.

## Results

### Semaphorin receptors Npn-1 and -2 and Plexin-A1, -A2 and -A4 are expressed by developing spinal motor neurons

**Figure 1:**
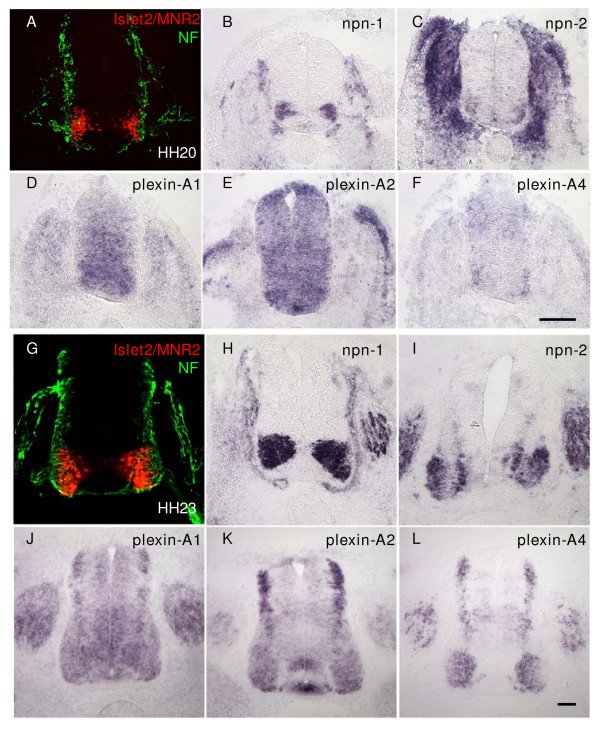
Expression of semaphorin receptors *Npn-1 *and *Npn-2*, and *Plexin-A1*, *-A2*, and *-A4 *by motor neurons in the chick embryo spinal cord. *In situ *hybridisations on transverse cryosections (20 μm) of chick embryo spinal cord (hindlimb level) from **(b-f) **HH stage 20 and **(h-l) **HH stage 23, show that *Npn-1 *(b,h), *Npn-2 *(c,i) and *Plexin-A1 *(d,j), *Plexin-A2 *(e,k), and *Plexin-A4 *(f,l) are all expressed by motor neurons. **(a,g) **Comparable sections from the same stage embryos immunolabelled with the motor neuron specific antibodies MNR2/islet2 (red) and counterstained with neurofilament antibody (NF; green). Bars = 50 μm.

Previously, we proposed that neural crest BC cells at the motor exit point (MEP) express repellent signals that help confine motor neuron somata within the chick embryo spinal cord as their axons first emerge into the periphery [18]. This requires that candidate receptors for putative repellents should be expressed by motor neurons from embryonic day (E) 2 when the first motor axons exit the spinal cord at the MEP. Available expression data in mouse and chick embryos suggested that prominent amongst these were the multimeric surface receptors for secreted class 3 semaphorins, neuropilins and/or plexins [13-15]. We therefore analysed in detail expression of chick neuropilins and the three known members of the chick Plexin-A family, Plexin-A1, -A2 and -A4 (there is no chick Plexin-A3 orthologue [15]) during the appropriate period. Our results confirm and extend those of Mauti *et al*. [15], showing dynamic patterns of expression in spinal motor neurons, with *Npn-1*, and *Plexin-A1 *and *-A2 *already present in progenitors at Hamburger Hamilton (HH) stage 18 (not shown). We found that Npn-2 is also expressed in motor neurons (or their progenitors) as early as HH stage 18, and in rostral parts of the embryo and throughout the embryo by HH stage19 (not shown). At HH stage 20 all *Npn *and *Plexin-A *receptors are expressed by motor neurons to some extent (Figure 1b–f), with *Npn-1 *most prominently and distinctly expressed. *Plexin-A1 *and *-A2 *are widely expressed throughout the ventral spinal cord at this stage. Within the spinal cord, *Npn-2 *and *Plexin-A4 *expression is restricted to laterally positioned motor neurons.

At the stage when we observed ectopic migration of motor neuron cell bodies following neural crest ablation (HH stage 23) [18], we found strong expression of npn-1 in most motor neurons (Figure 1h). *Npn-2 *is expressed in medial and lateral subsets of motor neurons but excluded from motor neurons located between these (Figure 1i). *Plexin-A1 *(Figure 1j) is more diffusely expressed throughout the ventral spinal cord, whereas *Plexin-A2 *(Figure 1k) and *-A4 *(Figure 1l) expression is restricted to lateral and medial motor pools, respectively. These expression studies show that all chick neuropilin and Plexin-A receptors examined could potentially regulate motor neuron cell body positioning.

### Targeting Npn-2 and Plexin-A2 by RNAi in the chick induces ectopic migration of motor neuron cell bodies

To assess the involvement of these receptors we applied a loss-of-function strategy in the chick, with vectors co-expressing shRNA and enhanced green fluorescent protein (EGFP), which marks those cells targeted by RNAi in an otherwise wild-type background [19].

We first knocked down *Npn-1 *and *Npn-2*, using shRNA vectors described previously [19,20]. We used an electroporation protocol that targets the plasmid DNA towards the ventral neural tube (for details see Materials and methods and Additional file 1). By doing so, effects on migration of the neural crest derivatives, including BC cells, can be excluded, an important consideration since both Npn-1 and Npn-2 have been implicated in neural crest migration [21,22].

**Figure 2:**
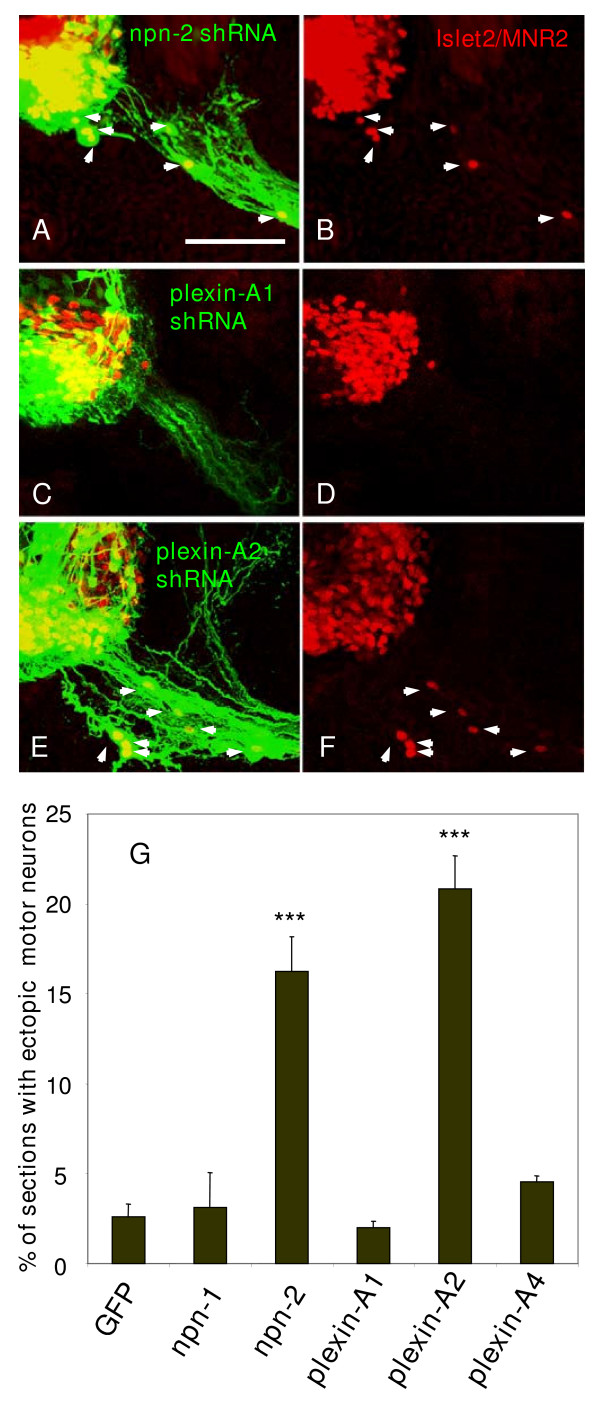
shRNA mediated selective knockdown of *Npn-2 *or *Plexin-A2 *mRNAs in the chick ventral spinal cord induces ectopic positioning of motor neurons. **(a-f) **Confocal micrographs of transverse vibratome sections (75 μm) of HH stage 24 embryo spinal cord 2 days after electroporation in the ventral neural tube. MNR2/Islet2 positive ectopic motor neurons (red; white arrows) are found in embryos electroporated with shRNA-EGFP vectors specific for *Npn-2 *(a,b) and *Plexin-A2 *(e,f), but not for *Plexin-A1 *(c,d). The presence of the shRNA vector is indicated by EGFP expression (green) such that MNR2/Islet2 positive motor neuron somata in ectopic positions are yellow (arrows in (a,e)). Bar = 150 μm. **(g) **Histogram showing percentage of HH stage 24 embryo sections containing dual labelled EGFP and MNR2/Islet2 positive ectopic motor neurons after ventral electroporations at HH stage 12–15 with shRNA-EGFP vectors targeting *Npn-1, Npn-2, Plexin-A1, Plexin-A2 *and *Plexin-A4*, or EGFP control vector. Only those shRNA constructs targeting Npn-2 and Plexin-A2 induced ectopic positioning of motor neurons. ***P < 0.001; two-tailed *t*-test.

We quantified the effect of shRNA-EGFP electroporation, compared to that of EGFP only, by counting ectopically positioned, EGFP-positive motor neurons. These were localised outside the spinal cord, along the ventral roots, and were characterised by their large soma linked to both trailing and leading EGFP-positive processes. Their identities were confirmed by dual labelling with antibodies to the transcription factors Islet-2 and motor neuron restricted protein (MNR)2. In our initial experiments with *Npn-2 *specific shRNA, we consistently observed ectopic EGFP/Islet-2/MNR-2 positive motor neurons in presumptive white matter and ventral roots (Figure 2a,b), but not with *Npn-1 *specific shRNA (Figure 2g). We subsequently generated four more vectors targeting Npn-2 and screened these for the potential to reduce *Npn-2 *expression in a co-expression assay [19]. One of these constructs, *Npn-2 *E shRNA, was as efficient as *Npn-2 *B shRNA in reducing Npn-2 expression (Additional file 2) and also consistently induced ectopic motor neurons, when electroporated in the ventral neural tube (Additional file 3).

Neuropilins do not signal independently but upon ligand binding associate with other molecules to confer an intracellular signal. Members of the Plexin-A family in particular have been implicated as obligate co-receptors [23] (for a recent review, see [24]). Therefore, we generated shRNA vectors targeting the three known members of the chick Plexin-A family, Plexin-A1, -A2 and -A4 (for details on shRNA vectors see Materials and methods and Additional file 4). When we screened these *Plexin *shRNA constructs for the potential to induce ectopic migration of motor neurons, one shRNA vector, targeting plexin-A2 (*Plexin-A2*A shRNA), substantially and selectively knocked down expression in motor neurons and triggered ectopic migration of motor neurons (Figure 2e,f; Additional files 5 and 6). In contrast, those vectors targeting Plexin-A1 (Figure 2c,d) or -A4 (not shown) that efficiently reduced expression of their target genes (Additional file 5) had no effect on motor neuron cell body positioning (Figure 2g and Additional file 3).

We constructed three more shRNAs targeted at Plexin-A2 and one of these, *Plexin-A2*D, also generated many ectopic motor neurons (Additional file 3).

In conclusion, quantitative analysis of these loss-of-function studies (Figure 2g) clearly identified Npn-2 and Plexin-A2 as receptors for ligands regulating motor neuron cell body position.

### Ectopic motor neurons are found in Npn-2 null mice

**Figure 3:**
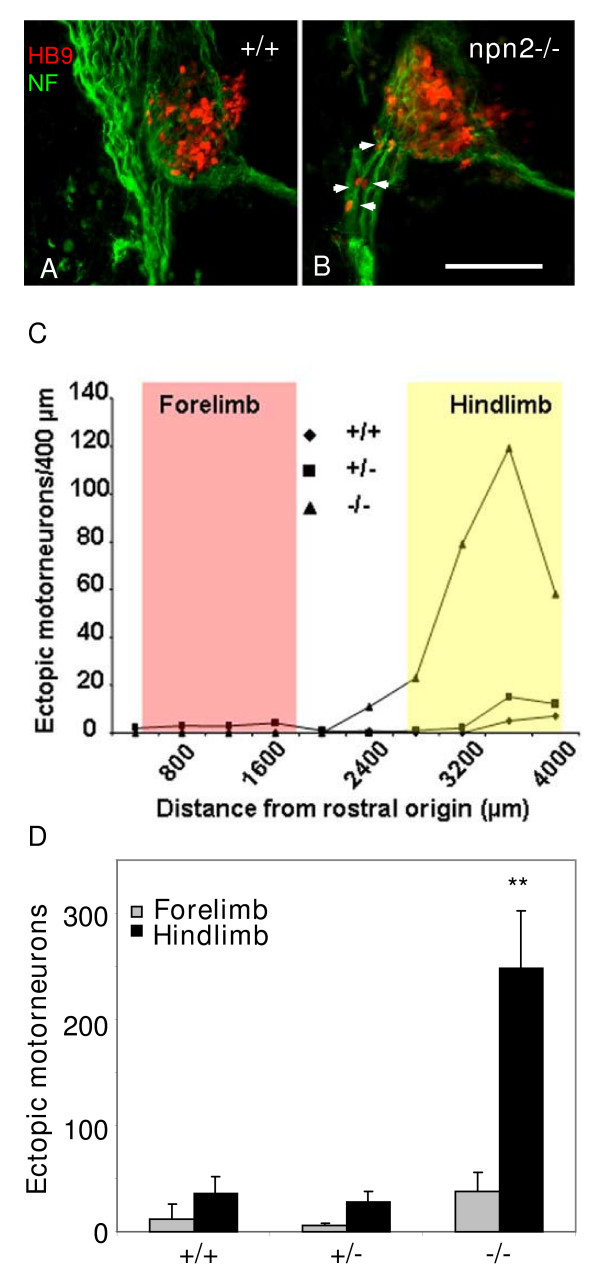
Motor neurons migrate to ectopic positions in *Npn-2 *null mice. **(a,b) **Dual immunostaining of transverse cryosections (20 μm) of E11.5 wild-type (a) or *Npn-2 null *(b) littermate mouse embryo spinal cord with antibodies against HB9 (red) or neurofilament (NF; green) reveal numerous HB9-positive ectopic motor neurons located in the marginal zone and ventral nerve root in mutant but not wild-type embryo sections ((b) white arrows). Bar = 150 μm. **(c) **A quantitative analysis of the distribution of HB9-positive ectopic motor neurons along the rostro-caudal axis of *Npn-2 *null (triangles) or heterozygous (squares) and wild-type (diamonds) littermate embryos indicates that the number of ectopic motor neurons is normal at forelimb level (red box) but peaks at hind limb level (yellow box). **(d) **A comparison of pooled and averaged number of HB9-positive ectopic motor neurons in the anterior (forelimb containing half) and the posterior (hindlimb containing half) of the trunk, for E12.5 *Npn-2 *wild-type, heterozygous and null littermate embryos (n = 4). Consistent with the quantitative analysis shown in (c), there is a significant increase in ectopic motor neurons in the hindlimb region of *Npn-2 *null mice. **P ≤ 0.01; two-tailed *t*-test.

In order to test whether this mechanism was conserved in a mammalian species, we examined *Npn-2 *null mice for evidence of ectopically positioned motor neurons. *Npn-2 *mutant mice have been analysed extensively in a number of studies of axon guidance phenotypes [13,25-27] and neuron migration [16,22,28] but defects in spinal motor neuron migration have not been reported. Yet we identified large numbers of ectopically positioned HB9-positive motor neuron cell bodies in *Npn-2 *null mouse embryos (Figure 3b). Interestingly, this phenotype appeared to be confined to more caudal regions of the trunk, prompting an analysis of the incidence of ectopic motor neurons throughout the rostro-caudal axis. A typical result of such an analysis in serial cryosections taken throughout the trunk revealed ectopically positioned HB9-positive motor neurons were largely restricted to the hindlimb level (Figure 3c). At forelimb and thoracic levels there was no significant increase above the background level in wild-type littermate embryos. The pooled results of several E12.5 embryos analysed in this manner (n = 4 for each genotype) confirmed the presence of large numbers of ectopic motor neurons in the hindlimb region of *Npn-2 *null mice (Figure 3d).

Although the outcome of targeted *Npn-2 *RNAi in the chick spinal cord indicates that the ectopic motor neuron phenotype could be a cell-autonomous effect, it remained a possibility that a reduced prevalence of BC cells at the MEP might contribute to the phenotype in *Npn-2 *null mice since the absence of Npn-2 leads to aberrant neural crest migration [22]. Therefore, using a specific marker for BC cells (Egr2/Krox20 [29]) we tested for the presence of these cells in *Npn-2 *null mouse embryos. The results (Additional file 7) show that at hindlimb level there was no obvious difference in egr2 expression at the MEP between *Npn-2 *null mice, and heterozygous and wild type littermates. Since in earlier grafting experiments in chick embryos we showed that small numbers of BC cells at the MEP were sufficient to confine motor neuron soma within the spinal cord [18], this supports the idea that the ectopic migration of motor neurons in *Npn-2 *null mice is a cell-autonomous effect.

### The Npn-2 ligands Sema3B and Sema3G and the Plexin-A2 ligand Sema6A are expressed by BC cells

**Figure 4:**
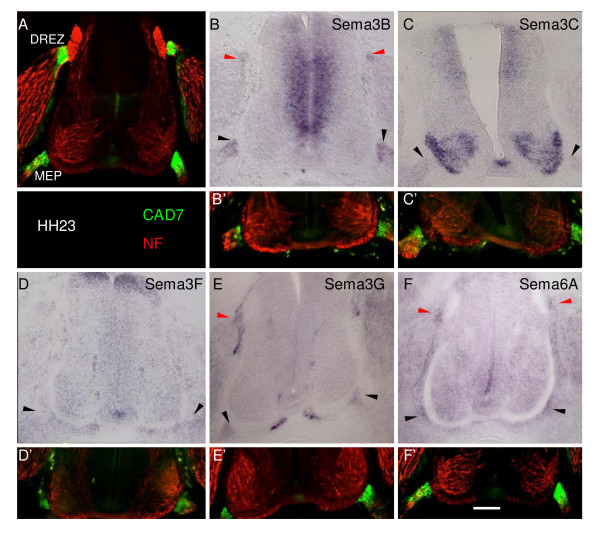
Expression of semaphorin genes in the trunk reveals putative repellent ligands are localised to ventral BC cells. **(a-f') ***In situ *hybridisations of HH stage 23 chick embryo transverse cryosections (b-f), in parallel with cad7(green) and neurofilament (NF; red) antibody staining on adjacent sections (a,b'-f') show that cad7-positive BC cells at the spinal cord DREZ (red arrows) and MEP (black arrows) express *Sema3B *(b), *Sema3G *(e) and *Sema6A *(f). In contrast, *Sema3F *(d) is not expressed at the MEP. Prominent expression of *Sema3C *(c) in motor neurons contrasts with a weak signal in ventral nerve roots. Bar = 100 μm.

When expression patterns of potential Npn-2 ligands in the chick were examined we found that Sema3B and Sema3G [30] are distinctly expressed by cad-7 expressing BC cells (Figure 4a) both at the dorsal root entry zone (DREZ; Figure 4b,b',e,e', red arrows) and the MEP (black arrows). In contrast, neither Sema 3C, prominently expressed in motor neurons (Figure 4c,c'), nor Sema3F, expressed broadly within somites (Figure 4d,d'), were localised to BC cells.

The finding that knockdown of Plexin-A2 compared to Npn-2 gave rise to greater numbers of ectopic motor neurons suggested that its role was more extensive than as just a Npn-2 co-receptor. Since the repellent activity of the transmembrane semaphorin Sema6A is mediated in sympathetic axons and hippocampal mossy fibres by direct interactions with Plexin-A2 and -A4 [31,32] we next looked for evidence of its expression. We found that Sema6A is expressed by both dorsal and ventral BC cells (Figure 4f,f').

Thus, we conclude that BC cells express at least three candidate repellent ligands for receptors on motor neurons identified in the RNAi screen, Sema3B, Sema3G and Sema6A.

### Genetic ablation of the Npn-2 ligand Sema3B does not induce ectopic migration of motor neurons

When we targeted Sema3B expression in the chick embryo neural crest by RNAi, we found no increase in the number of ectopic motor neurons (data not shown). However, since only small numbers of transplanted BC cells at the MEP suffice to stabilise motor neuron cell body position in the crest-ablated chick spinal cord [18], the possibility remained that residual untransfected BC cells were sufficient to mask the effects of RNAi.

**Figure 5:**
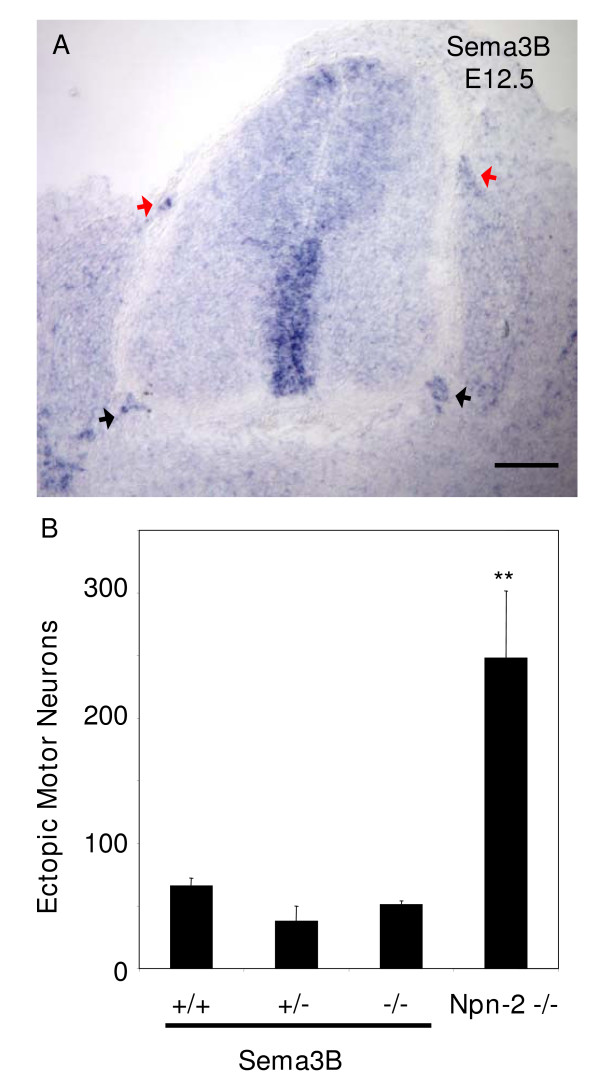
Genetic ablation of *Sema3B *in the mouse does not lead to ectopic positioning of spinal motor neurons. **(a) **As seen in the chick, in the E12.5 mouse trunk, *Sema3B *is expressed by BC cells located at the spinal cord DREZ (red arrows) and MEP (black arrows). Bar = 100 μm. **(b) **A quantitative analysis of the incidence of ectopic motor neurons at hindlimb level reveals that there is no significant difference between E12.5 *Sema3B *null mice and heterozygous and wild-type littermates (n = 3 each). This is in sharp contrast to E12.5 Npn-2 null mice, which is included for comparison (n = 4). **P ≤ 0.01; two-tailed *t*-test.

Therefore, we next analysed Sema3B mutant mice [27]. We first confirmed that Sema3B is also strongly expressed in mouse BC cells (Figure 5a). Analysis of the incidence of ectopic motor neurons throughout the rostro-caudal axis, however, did not reveal a difference between Sema3B mutant mice and heterozygous and wild-type littermates (Figure 5b). Therefore, loss of Sema3B expression despite its distinct expression by BC cells has no effect on positioning of motor neuron cell bodies.

### Loss of Sema6A expression in BC cells induces ectopic motor neuron positioning

**Figure 6:**
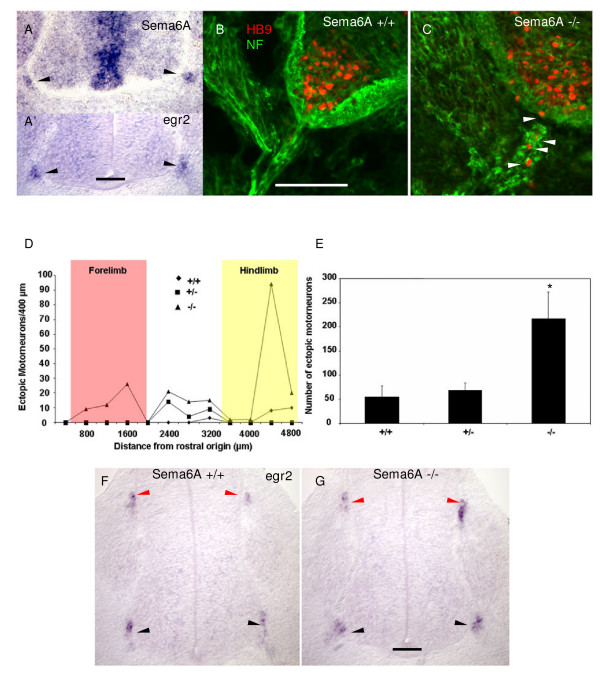
Genetic ablation of *Sema6A *in BC cells leads to ectopic migration of motor neurons. **(a) ***In situ *hybridisation shows expression of *Sema6A *in BC cells at the MEP (black arrows) in transverse cryosections of E12 mouse embryos. Expression of egr2/krox-20 in an adjacent section (a') confirms the signal in (a) corresponds to BC cells. Bar = 100 μm. **(b,c) **Dual immunostaining of transverse cryosections of E13.5 *Sema6A *mouse embryos. Compared with wild-type littermates (b) where HB9 positive motor neuron somata (red) are exclusively confined to the ventral spinal cord, in *Sema6A *null embryos (c) many motor neurons can be seen in ectopic positions in the neurofilament-positive (green) presumptive white matter and ventral nerve roots. Bar = 100 μm. **(d) **A quantitative analysis of the distribution of HB9-positive ectopic motor neurons along the rostro-caudal axis of E13.5 mouse spinal cord shows a distinct peak at hindlimb level (yellow box) but not forelimb level (red box) in null (triangles) compared with heterozygous (squares) and wild-type (diamonds) embryo littermates. **(e) **A comparison of the cumulative counts of HB9 positive ectopic motor neurons in sections from posterior trunk (hindlimb containing) region of E13.5 *Sema6A *wild-type, heterozygous and null embryos. Consistent with the quantitative analysis shown in (d), there is a significant increase (*p *= 0.03; two-tail *t*-test) in ectopic motor neurons in the hindlimb region of Sema6A null mice (n = 4 each). **(f,g) ***In situ *hybridisation for egr2/krox-20 on transverse cryosections (30 μm) of E11.5 mouse embryos at hindlimb level. The results show no obvious difference in *Egr2 *expression at the DREZ or MEP in wild-type (f) or *Sema6A *null mice (g), indicating that formation of boundary caps is not perturbed by genetic ablation of *Sema6A*. Bar = 100 μm. *P ≤ 0.05; two-tailed *t*-test

The finding of sema6A expression in chick motor exit point BC cells led us to examine expression in mouse. At E11.5 a distinct Sema6A signal is seen in motor exit point BC cells (Figure 6a, black arrowheads), in addition to prominent expression in the neural tube ventricular zone. Co-labelling with *Egr2 *(*Krox-20*) in an adjacent section (Figure 6a') confirmed expression was in BC cells. This prompted us to look for evidence of ectopic motor neuron positioning in Sema6A null mice [33]. Like in *Npn-2 *null mice, here we found numerous ectopic motor neurons in ventral nerve roots (Figure 6b,c). Also, in common with *Npn-2 *null mice, HB9-positive ectopic motor neurons are most prevalent at the level of the hindlimb (Figure 6d,e). An effect on neural crest migration and on the formation of boundary caps could account for this phenotype. However, as judged by *Egr2 *expression, there was no difference between Sema6A mutants and wild-type littermates (Figure 6f,g) in the arrangement of BC cells at the MEP and DREZ.

**Figure 7:**
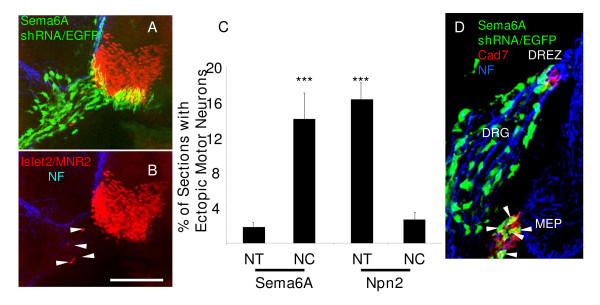
shRNA mediated knockdown of *Sema6A *in the neural crest, but not in the ventral spinal cord, induces ectopic motorneurons. **(a,b) **Confocal micrographs of vibratome sections of an HH stage 25 embryo, two days after electroporation in the neural crest with a *Sema-6A *shRNA-EGFP (green). Embryos were wholemount dual immunostained with antibodies against MNR2 and Islet2 (red) and counterstained with anti-neurofilament (NF, blue). MNR2 and Islet2-positive motor neurons (white arrowheads) can be seen in ectopic positions in the ventral roots but only when accompanied by a large number of *Sema-6A *shRNA expressing-EGFP-positive (green) neural crest derivatives. Note that EGFP-labelled cells are restricted to neural crest derivatives in the ventral root and that motor neurons, including those in ectopic positions are EGFP-negative. The small amount of EGFP labelling of structures in a ventro-medial position in the spinal cord corresponds to the axon projections of labelled commissural neurons in the dorsal spinal cord. Bar = 100 μm. **(c) **Quantitative analysis of the prevalence of ectopic motor neurons after electroporation with *Sema-6A *shRNA-EGFP targeted towards either the ventral neural tube (NT) or dorsal neural tube (neural crest, NC) shows a distinct effect only for neural crest-electroporated embryos. ***P < 0.001; two-tailed *t*-test. Conversely, knockdown of *Npn-2 *expression in the neural crest does not induce ectopic motor neurons, unlike knockdown of *Npn-2 *in the ventral neural tube, as shown earlier (Figure 2). **(d) **Confocal micrograph of a transverse cryosection (20 μm) immunolabelled with cad7(red) and neurofilament (blue) of an HH23 embryo 2 days after neural crest electroporation of *Sema6A *shRNA. GFP-positive neural crest cells targeted with *Sema-6A *shRNA populate the dorsal root ganglion (DRG), the DREZ and the MEP where they express high level of cad7 (red; white arrows), indicating that *Sema6A *shRNA does not interfere with boundary cap formation.

Since at later stages in the mouse *Sema6A *is expressed not only in BC cells but also in spinal motor neurons [14] (data not shown), it could not be excluded that the effect on motor neuron positioning in *Sema6A *null mice was due to its absence from motor neurons. Therefore, we next tested in chick embryos the effects of targeted knock down of Sema6A, either ventrally in motor neurons, or dorsally in the neural crest (Additional file 1). We found a significant increase in ectopic motor neurons, but only when *Sema6A *shRNA was introduced in the neural crest (Figure 7a,b), not in the ventral neural tube (Figure 7c). Knockdown of Npn-2 in the neural crest or ventral neural tube had the converse effect, with ectopic motor neurons found only after expression of Npn-2 shRNA in the ventral neural tube (Figure 7c). Finally, we analysed the effect on boundary cap formation of Sema6A knockdown in the crest. The data show that *Sema6A *shRNA-expressing neural crest cells are normally positioned at the MEP and these correspond to cad7-positive BC cells (Figure 7d, white arrows). Together, these results support the idea that the effect of *Sema6A *loss of function on motor neuron positioning is explained by loss of its expression in BC cells, disrupting a putative interaction with motor neurons.

### Targeting of the MICAL3 flavoprotein monooxygenase by RNAi in the chick induces large numbers of ectopic motor neurons

Having demonstrated that cell surface molecules involved in semaphorin signalling are implicated in regulating motor neuron cell body positioning, we next looked for possible common downstream cytoplasmic mediators. Members of the MICAL (molecule interacting with CasL) family of flavoprotein monooxygenases [34,35] are among an increasing number of molecules implicated in downstream semaphorin signalling. To investigate the role of MICALs in motor neuron cell body positioning, we performed RNAi on its chick orthologues.

**Figure 8:**
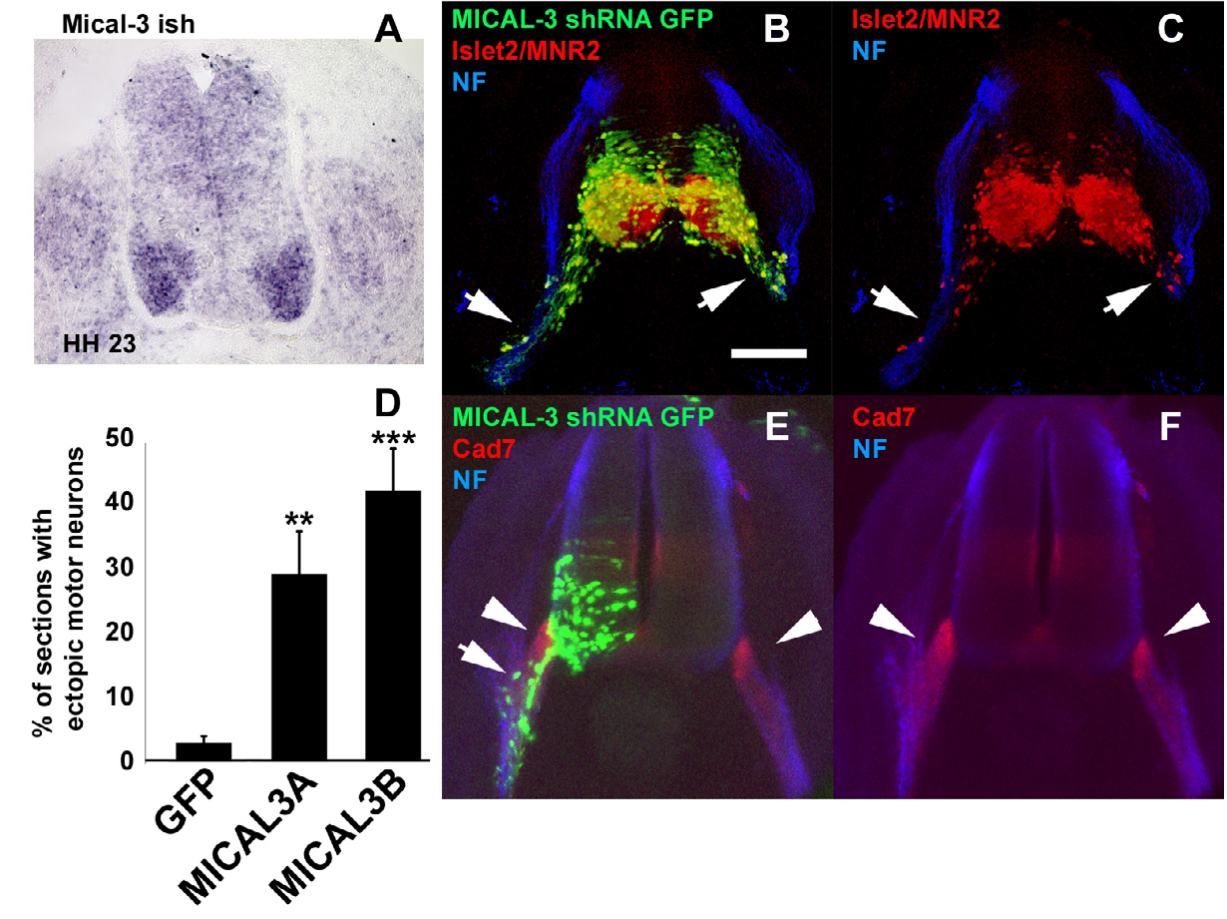
Knockdown of *MICAL3 *by ventral targeted shRNA-EGFP induces large scale ectopic migration of spinal motor neurons. **(a) ***In situ *hybridisation on HH stage 23 chick embryo transverse cryosections shows distinct expression of *MICAL3 *in motor neurons. **(b,c) **Targeting of *MICAL3 *shRNA-EGFP in the ventral spinal cord induces large scale ectopic migration of dual labelled GFP-positive (green (b)) Islet2/MNR2 positive (red (b,c)) motor neuron somata into ventral root (white arrows in (b,c)). The section was counterstained with anti-neurofilament (NF; blue) to locate the dorsal and ventral roots and show the specific, ventral targeting of the electroporation. **(d) **The percentage of vibratome sections from HH stage 24 embryos containing dual labelled EGFP- and MNR2/Islet2 positive ectopic motor neurons out of the total of EGFP-positive sections, after ventral electroporations with two shRNA-EGFP constructs *MICAL3*-A and -B, is significantly higher than when EGFP alone was electroporated. ***P < 0.001; two-tailed *t*-test. **(e,f) **Cad-7 expressing BC cells at the MEP (red; white arrowheads) are unaffected by *MICAL3 *shRNA knock down in the ventral spinal cord (green in (e)) that induces large scale motor neuron migration (arrow in (e)). Bar = 100 μm.

We identified three MICALs in the chicken genome, *MICAL1 *on chromosome 26, *MICAL2 *on chromosome 5 and *MICAL3 *on chromosome 1. In contrast to *MICAL2 *and 3, *MICAL1 *sequences are not present in a comprehensive chick expressed sequence tag (EST) expression database [36], suggesting it is not highly expressed. *In situ *hybridisation expression analysis for *MICAL3 *showed that this gene was distinctly expressed by motor neurons (Figure 8a; a more comprehensive study on chick MICALs is in progress; MV, unpublished results). We constructed shRNA expressing vectors targeting the predicted *MICAL3 *mRNA and electroporated these in the chick embryo ventral spinal cord. We found that *MICAL3 *shRNA causes substantial numbers of motor neurons to exit the spinal cord by translocating along their axons in the ventral roots (Figure 8b,c). The magnitude of the effect of MICAL3 shRNA (Figure 8d) is considerably larger than that found with *Npn-2 *or *Plexin-A2 *shRNA, approaching the numbers we observed after complete surgical neural crest ablation in chick [18]. Ectopic migration following loss of MICAL3 in motor neurons occurs despite the presence of BC cells at the MEP (Figure 8d,e). Together, these results support the conclusion that the cytoskeletal re-organisation that uncouples somal migration from axon elongation in motor neurons involves multiple semaphorin-induced signals that converge on a common cytoplasmic mediator.

## Discussion

### Motor neuron somal positioning is the result of a local response to a local signal

The migration of neurons to their final destinations is a key process in the development of the nervous system. This process must be coordinated with the establishment of connectivity and requires differential responses of distinct neuronal compartments to the multitude of guidance cues in the environment. This could be achieved in several ways: first by the sorting of compartment-specific receptors, such as at the axonal initial segment [37]; the selective accumulation of kinesin motors in axons versus dendrites [38]; by the localised intracellular content of modifiers, such as small cyclic mononucleotides [39]; and lastly by the different extracellular environments in which each compartment is located. While this is obvious at the level of the growth cone, where localised signals lead to the re-organisation of the cytoskeleton and growth cone turning, an appealing idea is that the cell bodies of neurons are similarly responsive to cues in their local environment. The logistics of this problem is particularly challenging in the case of long distance projection neurons like somatic motor neurons. Their cell bodies begin to settle in position within the ventral spinal cord at an early phase of differentiation before their axons and dendrites make appropriate connections with their target cells.

In previous work we identified a key role for neural crest derived BC cells in the regulation of this process. In the absence of these cells, spinal motor neuron cell bodies follow their axons at the MEP and migrate into the periphery [18]. Here we have begun to identify the molecules involved in the interaction between BC cells positioned at the MEP and motor neurons that prevent somal migration out of the spinal cord. Using loss-of-function studies in chick and mouse, we present evidence for the involvement of members of the semaphorin family in BC cells and their receptors on motor neurons. Loss of Npn-2, a receptor for class 3 semaphorins, in motor neurons leads to ectopic migration of these neurons, both in chick and mouse spinal cord, as does loss of the transmembrane semaphorin Sema6A in BC cells. RNAi studies in chick identified a role for Plexin-A2 in motor neurons, either as a transducing component in a signalling complex with the ligand binding receptor Npn-2, or as a direct receptor for Sema6A. The requirement for semaphorin-plexin signalling in cell body positioning is revealed by the potent effect of loss-of-function of MICAL3, a member of a conserved family of intracellular Plexin-A binding proteins that have been identified as key modulators of semaphorin-plexin signalling [34,35].

### Loss-of-function analysis in the chick with shRNA/EGFP vectors

We started our search for candidate molecules involved in the control of motor neuron migration at the MEP using an RNAi approach that we had developed in the chick, utilising a vector system co-expressing shRNA and EGFP [19]. We combined this loss-of-function approach with protocols for targeted expression of transgenes in chick embryos [40] (Additional file 1), which allowed us to selectively knock down expression of candidates in either the ventral neural tube, where motor neurons develop, or in the neural crest, the progenitors of BC cells. Thus, we were able to conclude that Npn-2 and Plexin-A2 are required in motor neurons, whereas Sema6A functions as a BC cell expressed ligand in helping stabilise motor neuron cell body position in the ventral spinal cord.

Since the emergence of RNAi as a powerful experimental tool, several pitfalls associated with this technique have become apparent. First, when using randomly selected small interfering RNA (siRNAs) duplexes or shRNAs, at least half of the sequences will not induce any reduction of their targets and less than 5% will reduce expression by 90% or more [41]. Therefore, we selected our RNAi targets using an algorithm for rational siRNA design (SFOLD [42]) that predicts target accessibility and applies empirical rules for functional siRNA duplexes [41]. Consequently, approximately one in three of our constructs proved effective, in that they significantly reduced expression of their intended targets as determined by an *in vitro *co-expression assay or by *in situ *hybridisation (Additional files 2, 5 and 6).

Second, off-target and non-specific effects have been widely reported [43-45] and are a serious concern when using RNAi. However, we are confident that the ectopic motor neuron phenotypes we report here are related to the loss-of-function of the intended targets of our constructs, as they are duplicated by constructs targeting the mRNAs on different sites (*Npn-2, Plexin-A2 *and *MICAL3*). During this study we tested a total of 38 shRNA hairpin constructs aimed at 10 targets for the induction of ectopic motor neurons after ventral electroporation. Even when considering our collection of shRNAs as random (yet they are all aimed at molecules involved in semaphorin signalling, and all of those are expressed in the ventral spinal cord to some extent), the probability of six hits appearing in three pairs is quite small (*p *= 0.0111; using an analysis derived from a hypergeometric test). Were we to expand our analysis with shRNAs aimed at truly random targets, the predicted outcome would be that the hit rate would decrease and, concomitantly, the significance of hits appearing in pairs would increase even further. This indicates that general non-specific or random off-target effects are a highly unlikely explanation for the set of data that we have obtained. Finally, the fact that we were able to confirm the roles for Npn-2 and Sema6A in the mouse reinforces the main conclusion from the chick RNAi work that multiple semaphorin-induced pathways are involved in maintaining the stability of motor neuron somal position.

### A role for semaphorin-plexin signalling from BC cells in the maintenance of motor neuron soma position is conserved in amniotes

Our loss-of-function studies in chick and mouse implicate two proteins in the control of motor neuron migration at the MEP. Npn-2 is required in motor neurons and Sema6A in BC cells. Sema6A and Npn-2 are not known to interact with each other. Therefore, we propose the involvement of at least two separate pathways mediating this mechanism, although, in contrast to Npn-1 [31,46], a contribution of Npn-2 to class 6 Sema signalling cannot formally be ruled out. Our more extensive studies in chick identified a role for Plexin-A2 in motor neurons. Consistent with its expression in motor neurons (Figure 1e,k), chick Plexin-A2 could function as a dual receptor for both class 6 and class 3 semaphorins. However, in the mouse we failed to detect significant expression of Plexin-A2 in motor neurons at any stage (results not shown). Considering there are three members of the Plexin-A family in chick, unlike in mouse, where there are four, differences in the individual contributions of the various members of the Plexin-A family to chick or mouse neural development are to be expected. The role of Plexin-A2 as a class 6 semaphorin receptor in chick motor neurons could be adopted by Plexin-A4 in the mouse, as this receptor is more strongly expressed by mouse spinal motor neurons (unpublished observations) and can function as a Sema6A receptor in mouse [32]. Plexin-A3, which is expressed by motor neurons in the mouse [47], has already been implicated as the preferred co-receptor for Npn-2 in mouse sympathetic neurons [48]. Clearly, our observations warrant further studies in *Plexin-A *mutant mice [31,32,47,49].

So far we have been unable to functionally implicate a class 3 semaphorin in the maintenance of motor neuron position, despite the strong evidence for involvement of Npn-2, and the distinct expression of the Npn-2 ligands Sema3B and Sema3G in BC cells. Since at least two known Npn-2 ligands are expressed by BC cells, a single hit loss-of-function approach may fail as a result of compensatory mechanisms. Experiments aimed at reducing the expression of Sema3B and Sema3G in chick neural crest simultaneously by RNAi have so far not demonstrated a significant increase in the numbers of ectopic motor neurons (not shown). However, considering we found that the ectopic motor neuron phenotype after surgical ablation is effectively rescued by small numbers of grafted BC cells [18], residual levels of repellent molecules at the MEP after partial knockdown are unlikely to produce a strong phenotype. Therefore, a thorough analysis of the role of Sema3B and -3G in motor neuron somal positioning at the MEP will probably require creating double mutants in mouse. Such an analysis will be feasible when Sema-3G mutant mice become available.

It has been proposed, based on their complementary expression patterns, that semaphorins and their receptors could play a role in motor pool sorting in the embryonic mouse ventral spinal cord [14]. We cannot presently exclude the possibility that some of our loss of function experiments could directly perturb interactions between neighbouring motor neurons, leading to a possible disruption of pool cohesion and motor neuron positioning. However, this seems unlikely for two reasons. Firstly, no evidence was found in earlier studies of semaphorin and *Npn-2 *null mutants of any effects on motor pool organisation [13]. Secondly, in our chick electroporation experiments where motor neurons were targeted, we rarely saw HB9-positive ectopic cells that were not GFP-positive (less than 1% of the ectopic motor neurons were GFP-negative; for example, see Figure 2a,b,e,f). Moreover, targeted disruptions by electroporation of transgenes in spinal motor neurons engineered to induce pool sorting errors lead to minor cell body displacements within the ventral spinal cord [10] rather than the large scale ectopic migration we found.

An unexpected finding is the restriction of an ectopic motor neuron phenotype to caudal-most regions of *Npn-2 *and *Sema6A *null mouse embryos. It seems unlikely that BC cells in the mouse confine motor neurons to the spinal cord only at caudal levels since we have previously described the occurrence of ectopic motor neurons at rostral levels in the Splotch (*Pax3*) mutant mouse [18], as well as in *Sox10 *null mouse embryos (Vermeren and Cohen, unpublished). In both mutants, embryos lack BC cells at rostral levels and their ventral nerve roots contain numerous ectopic motor neurons. One possible explanation is that this could reflect level-specific differences in expression of both Npn-2 and Sema6A in spinal motor neurons and BC cells. These differences clearly exist in the mouse [14] (unpublished observations) but not to such an overt extent that could explain the restricted localisation of this phenotype. Another explanation may lie in the temporal gradient of maturation along the rostro-caudal neuraxis, making the more immature hindlimb motor pools more susceptible to perturbations. This is perhaps hinted at by the higher background levels of ectopically positioned motor neurons we saw in caudal-most regions of wild-type mouse embryos (Figures 3c and 6d).

Further evidence for the involvement of semaphorin-plexin signalling in motor neuron somal settling is the potent effect of MICAL3 loss-of-function in the chick. MICALs are a family of conserved cytosolic signalling proteins known to interact directly with Plexin-As and cytoskeletal or cytoskeleton associated molecules (for a review, see [50]). *Drosophila *MICAL was identified in a yeast-two-hybrid screen using D-Plexin-A cytoplasmic domain as bait and the D-MICAL loss of function (LOF) mutant has severe motor axon guidance defects, similar to those found in *Sema-1a *and *PlexA *mutants [34]. In relation to axon guidance, MICALs have been implicated in Plexin-A signalling only. Furthermore, D-MICAL does not interact with PlexB [34]. Thus, the evidence so far suggests that MICALs might be specific mediators of semaphorin-plexin-A signalling. Experiments with a pharmacological inhibitor of flavoprotein monooxygenases, epigallocatechin gallate (EGCG), suggested that axon repulsion by class 3 semaphorins might depend on the oxidoreductase activity of MICALS [35]. EGCG did not inhibit the repulsive effects of a number of other ligands, including Sema6A. However, this does not rule out that MICALs are intermediaries in the signalling induced by Sema6A and other repellents, independent of their oxidoreductase activity.

Our loss-of-function studies in the chick ventral spinal cord suggest a ranking of targets, according to the severity of the phenotype, MICAL3 LOF > Plexin-A2 LOF > Npn-2 LOF (quantified in Additional file 8). This ranking is consistent with a signalling hierarchy, where a diverse set of semaphorins (Sema3B, 3G, 6A) interacts with a number of signalling receptors or receptor complexes (Plexin-A2 and Npn-2/Plexin-A). The signals emanating from these receptors then converge on a common intermediate (MICAL3). The effect of MICAL3 LOF approaches the severity of the ectopic motor neuron phenotype observed after surgical neural crest ablation [18]. This suggests that, if they exist at all, the contributions of parallel, MICAL3-independent pathways will be necessarily minor. We are currently investigating the role of the two other chick MICAL orthologues, alongside other intermediary molecules known to be involved in semaphorin-plexin signalling pathways, such as CRMPs [51-54].

### Future directions: linking interactions at the cell surface with the regulation of somal translocation

**Figure 9:**
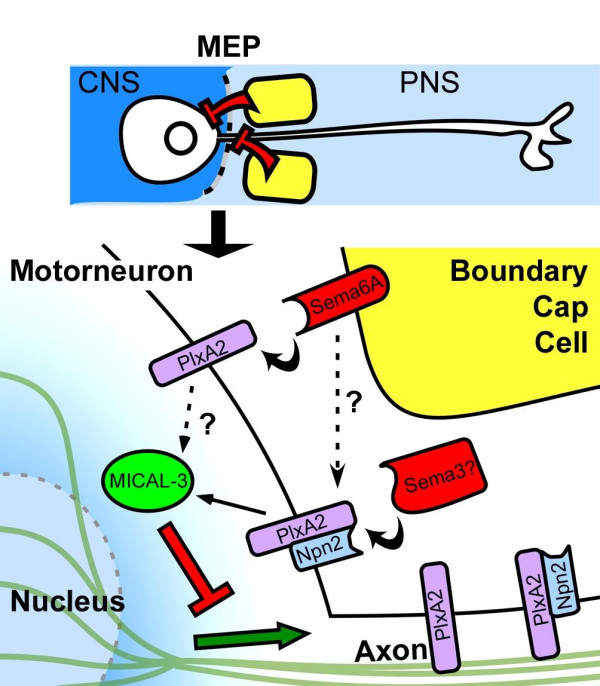
Summary of the pathways in motor neurons implicated in mediating a BC cell derived repellent signal that stops somal migration. Boundary cap cells (yellow) located at the MEP express repellent signals (red) that act either directly on motor neuron cell bodies or retrogradely via their axons to disengage somal migration from axon extension. Our data suggest that these signals comprise a combination of class 3 semaphorins and Sema6A (red). Plexin-A2 (purple) expressed by the motor neurons can function as a dual receptor for class 3 and class 6 semaphorins, the former in conjunction with Npn-2 (blue). In this scheme, MICAL3 (green), by linking Plexin-A to the cytoskeleton, is a key downstream mediator of the somal stabilising signal. As a result of cytoskeletal re-organisation, the force (green arrow) exerted from the axonal growth cone that leads to somal translocation in migrating neurons is disrupted.

The saltatory motion of migrating immature neurons is dependent on a microtubule perinuclear cage that drags the nucleus and soma unidirectionally [55,56]. Several intracellular proteins are known to be involved in this process and their loss leads to pathologies characterised by ectopic migration, such as lissencephalies and epilepsy [4]. We propose that the effect of the localised semaphorin-PlexinA2-MICAL3 signal that we have identified is to neutralise or provide an opposing force to the axon-driven perinuclear cage drag (Figure 9).

Little is known about exactly what MICALs interact with except an affinity for the cytoskeleton [57]. However, MICALs have clearly been implicated in class3 semaphorin signalling, which is best known for its role in axon guidance and cytoskeletal remodelling following growth cone turning (for reviews, see [24,58]). This process relies notably on type II myosin [59], the over-activation of which leads in the fly to the ectopic migration of photoreceptor cell bodies from the single layered eye disk along the optic nerve as far as the optic lobe [60]. In growth cones, type II myosin generates a force that underlies the retrograde flow of actin. This force is antagonised by dynein [61], part of the dynein/dynactin negative end microtubule motor transport complex. Interestingly, null mutation of p150 glued, a subunit of dynactin, in *Drosophila *photoreceptors also leads to their ectopic migration into the optic lobe [62,63]. There are clear parallels between these observations and our results: in both scenarios normally unipolar neurons become bipolar through the perturbation of a signal (or its effectors) that antagonises perinuclear cage drag. Recently, it was shown that knock-down of both cytoplasmic dynein and myosin II stalls glial-guided somal migration of neuronal precursors [64]. These findings resonate with our work but leave open the question how these changes are regulated at the cell surface of neurons. We suggest that for motor neurons, semaphorin signalling from BC cells triggers cytoskeletal changes that lead to migration arrest. When cell body migration stops, the stabilisation of neuronal cell body position requires that uncoupling of the perinuclear cage drag be sustained throughout the lifetime of the neuron. Since BC cells are a transient population [65], what constraints prevent motor neuron somata from emigrating at later stages? The most likely explanation is that as motor neurons mature, their polarised structure becomes stabilised by the onset of cadherin-mediated interactions with neighbouring motor neuron somata [10], under the influence of a variety of environmental factors, most importantly innervation from muscle targets, primary sensory afferents and the corticospinal tract.

## Conclusion

We have found evidence that semaphorin-mediated interactions between BC cells and immature spinal motor neurons regulate somal positioning by counteracting an intrinsic tendency of motor neuron cell bodies to translocate along their axons into the PNS. These data support a model in which BC cell semaphorins signal through Npn-2 and/or Plexin-A2 receptors on motor neurons via MICAL3, a cytoplasmic effector, to trigger cytoskeletal reorganisation. This leads to the irreversible uncoupling of somal migration from axon elongation at the MEP (Figure 9).

## Materials and methods

### Animals

All animal procedures were performed in accordance with institutional guidelines. *Npn-2 *[66], *Sema3B *[27] and *Sema6A*-deficient mice [17,67] were bred and genotyped as described.

### *In ovo *electroporation and shRNA constructs

Targeted electroporation towards the ventral neural tube was essentially performed as described by [40], using an Intracept TSS10 pulser (IntraCell Shepreth Herts, UK) or a Grass-Telefactor SD9 stimulator and 0.25 mm silver electrodes. Plasmid DNA, resuspended in TE buffer at 1 mg/ml with 0.2% Fast Green FCF, was injected in the neural tube of embryonic day 2 (HH stage 10–15) embryos and 3–5 square pulses of 10–12 V were applied with the positive electrode piercing the egg shell and positioned underneath the trunk and the bent, negative electrode above the embryo, parallel to the positive electrode.

For dorsal horn/neural crest targeting, the polarities of the electrodes were reversed and 2–3 square pulses of 7–10 V applied. To limit the size of the targeted area, the positive electrode was positioned perpendicularly to the negative electrode. As a consequence, expression in the dorsal neural tube is restricted to the equivalent of one or two somites, whereas EGFP expressing neural crest derivatives can be observed several somites away from the electroporation site (Additional file 1). The embryos were left to develop for a further two days post-electroporation (HH 22–25) before being fixed in 4% paraformaldehyde (PFA) for antibody staining and analysis. To facilitate the rapid generation of RNAi constructs, co-expressing shRNA and EGFP, we created the pCAβ-EGFPm5-mU6 vector. The *Apa*I site at position 864 in pCAβ-EGFPm5 [19,68] was removed by digestion with *Apa*I and filling in using T4 polymerase. Subsequently, a 376 bp cassette containing the mouse U6 promoter was cut from pBS/U6 (aka pSilencer1.0) [69] using *Bam*HI and inserted into the *Sal*I site at position 1 of the modified pCAβ-EGFPm5 vector, after blunt ending of the *Bam*HI and *Sal*I overhangs with T4 polymerase.

RNAi target sites of 21 nucleotides length were selected on the respective target mRNAs using the SFOLD algorithm [42,70] at. The specificity of the selected targets was checked by BLAST. Synthetic complimentary oligonucleotides containing hairpin sequences were designed using the Ambion pSilencer Insert Designer [71], using the default loop sequence of TTCAAGAGA. This oligonucleotide design attaches overhangs matching *Apa*I (GGCC) to the 3' end and *Eco*RI (AATT) to the 5' end of the bottom strand. The *Eco*RI overhang was omitted from the design in order to allow the annealed oligonucleotides to match the blunt end in the vector generated by *Eco*RV. Oligonucleotides were purchased from MWG Biotech AG (Ebersberg, Germany) or OPERON, (Cologne, Germany). Annealed oligonucleotides were cloned into the unique *Apa*I and *Eco*RV sites of the pCAβ-EGFPm5-mU6 vector. Details of the shRNA constructs used in this paper can be found in Additional file 4.

### Immunohistochemistry, microscopy and quantification of ectopic motor neurons

Whole-mount immunostaining of electroporated chick embryos was performed as described before [18]. Mouse monoclonal 3A10, anti-Islet-1/2 (39. 5D5), anti-Islet-2 (51. 4H9), and MNR2 (81. 5C10) were developed by Dr Thomas Jessell. These antibodies were obtained from the Developmental Studies Hybridoma Bank, developed under the auspices of NICHD and maintained by the University of Iowa, Department of Biological Sciences, Iowa City, IA, USA. Rabbit anti-neurofilament (NF1991) was purchased from Millipore (Watford, UK); rabbit anti-HB9 from Abcam (Cambridge, UK); rabbit anti-GFP from Invitrogen (Paisley, UK). Mouse anti-V5 was obtained from Sigma-Aldrich (Dorset, UK).

Post-staining, embryos were cleared in a glycerol series, embedded in 20% gelatin/PBS and sectioned on a Leica VT1000S vibratome. Sections (75 μm) were collected on glass slides and mounted with MOWIOL or Vectashield. For quantification of ectopic motor neurons, sections were viewed on a Zeiss Axioskop fluorescence microscope.

The effect of each shRNA-EGFP construct was quantified by counting the number of sections containing ectopic Islet-2/MNR2 positive motor neurons at the EGFP-positive electroporation site (for quantification, see Additional file 8). When the ventral spinal cord was electroporated, the ectopic neurons were EGFP-positive, and when the neural crest was electroporated, peripheral glia and BC cells were EGFP-positive. Since electroporation results in a mosaic embryo, there were instances where the region of the ventral spinal cord that was EGFP-positive contained few labelled motor neurons. As results were expressed as the number of sections containing EGFP-Islet2/MNR2 positive neurons over the overall number of sections containing EGFP, the proportions obtained represent an underestimate of the incidence of ectopic motor neurons. A minimum of six embryos were analyzed for each construct. Representative images were scanned on an Olympus Fluoview confocal laser scanning microscope connected to an Olympus AX70 research microscope. Images were processed using the software supplied by the manufacturer.

For analysis of ectopic motor neurons in mice, embryos were fixed for 2 h in 4% PFA on ice, followed by 2 × 30 minute washes in PBS. Subsequently, embryos were equilibrated in 30% sucrose in PBS, decapitated, the bodies cut in half at mid-trunk level and frozen in Optimal Cutting Temperature (OCT) compound. Serial sections of 20 μm thickness (approximately 20 sections per slide) were cut in the transverse plane on a Bright cryostat. Sections were subsequently dual-labelled with rabbit anti-HB9 and mouse monoclonal 3A10 antibodies and antibody binding visualised by incubating with appropriate fluorescent second antibodies. Slides were coded before the incidence of ectopic motor neurons (that is, HB9 positive neurons in the presumptive white matter or within ventral roots) was quantified by an investigator who was unaware of the code. Images were collected using a Zeiss Axioskop fluorescence microscope connected to a Spot CCD camera.

### *In situ *hybridisation

Digoxigenin-labelled antisense copy RNA was transcribed from linearised cDNA templates and hybridised with 20 μm cryostat sections, as described previously [72,73]. A cDNA probe for chick *Plexin-A1 *[74] was a gift from Dr Britta Eickholt; chick *Npn-1 *from Dr Jon Raper; chick *Npn-2 *from Dr Gera Neufeld; chick *Sema3F *from Dr Yuji Watanabe; chick *Plexin-A2 *(ChEST887n12), *Plexin-A4 *(ChEST202o14), chick *Sema3B *(ChEST771a21), chick *Sema3C *(ChEST997l4), chick *Sema6A *(ChEST667n18), chick *MICAL2 *(ChEST58n1), and chick *MICAL3 *(ChEST59i4) were obtained from the BBSRC chick EST database [36] via the MRC Geneservice. Chick *Sema3G *cDNA (clone pgf1n.pk012.d10) was obtained from the chick EST database from the University of Delaware. Mouse *Sema3B *cDNA was a gift from Dr Andreas Püschel; mouse *Sema6A *cDNA from Dr Alain Chedotal. Mouse *Egr-2 *(*Krox20*) cDNA was a gift from Dr Piotr Topilko.

Images were viewed using Nomarski optics on a Zeiss Axioskop and images stored on a Spot 2 DCC camera.

## Abbreviations

**CRMP : **Collapsin Response Mediator Protein

**Cxcr **: Chemokine C-X-C motif Receptor

DREZ : Dorsal root entry zone; 

EGFP : Enhanced green fluorescent protein;

Eph : Erythropoietin-producing human hepatocellur

EST : Expressed sequence tag; 

**ETS : **E-Twenty-Six

HH : Hamburger Hamilton; 

LOF : Loss of function; 

MEP : Motor exit point; 

MICAL : Molecule interacting with CasL; 

MNR : Motor neuron restricted protein; 

Npn : Neuropilin; 

PBS : Phosphate-buffered saline; 

PLM : Posterior lateral motor; 

PNS : Peripheral nervous system; 

RNAi : RNA interference; 

shRNA : Short hairpin RNA; 

siRNA : Small interfering RNA.

## Competing interests

The author(s) declare that they have no competing interests.

## Authors' contributions

JC, RB and MV conceived and designed the study, along with the help of NK in the lab of JC. All of these authors provided intellectual input for the study, data interpretation and helped write the manuscript. MV and RB performed the targeted *in ovo *electroporation studies and immunolabelling analysis in chick. The construction of the pCAβ-EGFPm5-mU6 vector and shRNA plasmids were performed by NK and RB. Antibody staining and immunolabelling analysis of mouse embryos was performed by NK, as were *in situ *hybridisation experiments in mouse and chick. Generation of MICAL specific shRNAs and all related functional studies in chick were designed and executed by MV. The breeding of neuropilin-2 knock-out mouse embryos was performed by WA. Semaphorin 6A mutant mouse embryos were generated by GEL in the lab of KJM. WA, GEL and KJM also participated in manuscript preparation. All authors have read and approved the final manuscript.

## Supplementary Material

Additional file 1 Targeted delivery of EGFP expression plasmids by selective electroporation towards either the ventral neural tube or the neural crest. Confocal micrographs of transverse vibratome sections (75 μm). **(a) **An example of a HH23 chick embryo electroporated in the ventral neural tube with the pCA-EGFPm5-mU6 plasmid, as detailed in the Materials and methods. Motor neurons are labelled with a mixture of anti- MNR2 and Islet-2 antibodies (red). Note that virtually all islet-2/MNR2 positive motor neurons (MN) in this section appear yellow, indicating co-expression of EGFP. No EGFP expression is seen outside the neural tube. **(b) **The plasmid was targeted towards the dorsal neural tube. The section shown was taken several somites away from the electroporation site and shows EGFP expression in neural crest derivates only (dorsal root ganglion (DRG), sympathetic ganglia (Sn) and glia in ventral (VR) and dorsal (DR) spinal nerve roots). The anti-Islet-1,2 antibody (red) labels motor neurons, DRG neurons, sympathetic neurons and dorsal horn interneurons. Note the complete absence of EGFP expression within the spinal cord. Bar = 100 μm. Click here for file

Additional file 2 *Npn-2 *B and *Npn-2 *E shRNA reduce expression of V5-tagged cNpn-2 in HEK cells. Vectors co-expressing EGFP and shRNA targeted at *Npn-2 *were co-transfected with pcDNA3.1-V5-c*Npn-2 *[19] at a ratio of 5:1. At 2 days post-transfection, cells were fixed and stained with V5 antibodies. The data show efficient knockdown of V5-tagged Npn-2 with *Npn-2 *B and *Npn-2 *E shRNA. By contrast, the *Npn-2 *F shRNA construct is ineffective. Bar = 25 μm. Click here for file

Additional file 3 Screen of shRNA vectors targeting Npn or Plexin-A receptors in the chick for the ability to induce ectopic motor neuron migration. The prevalence of ectopic motor neurons after ventral electroporation of shRNA vectors targeting plexin-A and neuropilin receptors in the chick was assessed. In a first round of analysis, three different vectors were tested for each Plexin-A target along with validated npn-1 and *Npn-2 *shRNA vectors. After obtaining positive results for *Npn-2*B and *Plexin-A2*A shRNA, a second batch of triplicate vectors targeting Npn-2 and Plexin-A2 was tested. Of these vectors *Npn-2 *E and *Plexin-A2*D shRNA also induced ectopic motor neurons, whilst Npn-2 C, -2D, -2F and Plexin-A2E and -A2F constructs were ineffective. ***P < 0.001; two-tailed *t*-test. Click here for file

Additional file 4 Table of shRNA target sequences and SFOLD scores (on a 0–20 scale). Note that the *Npn-1*B and *Npn-2 *B shRNAs we published previously have a relatively low SFOLD score, yet efficiently reduced expression of their targets [19]. Click here for file

Additional file 5 Confirmation of knockdown of *Plexin *mRNAs by ventral targeted shRNA in the chick spinal cord. Embryos were electroporated at E2 (HH stage 13–15) in the ventral neural tube with shRNA vectors targeting **(a,b) ***Plexin-A1*, **(c,d) ***Plexin-A2 *or **(e,f) ***Plexin-A4*. The effect of each shRNA on expression was assessed after 48–54 hours (HH 23–24) by *in situ *hybridisation on transverse cryosections of hindlimb region spinal cord (20 μm) (b,d,f). Bar = 100 μm. The data show a clear reduction of the *in situ *signals of the targeted genes in the electroporated areas (enclosed by dashed lines), compared to corresponding regions on the un-electroporated, contralateral side. Click here for file

Additional file 6 Specificity of knockdown by *Plexin-A2 *shRNA. An embryo was electroporated at E2 (HH stage 13–15) in the ventral neural tube with an shRNA vector targeting *Plexin-A2*. The effect at HH stage 23 of shRNA electroporation **(a) **on expression of **(b) ***Plexin-A1*, **(c) ***-A2 *and **(d) ***-A4 *was assessed on adjacent transverse cryosections of hindlimb region spinal cord (30 μm). *Plexin-A2 *shRNA was effective in reducing *Plexin-A2 *mRNA (c) (marked knockdown evident in regions enclosed by dotted lines in (a,c)) but was without effect on expression of *Plexin-A1 *(b) and Plexin-*A4 *(d). **(e) **To more precisely map the region of *Plexin-A2 *shRNA expression onto the *Plexin-A2 *gene expression pattern, EGFP was visualised by GFP antibody labelling (green) post hybridisation on a section from the same embryo as above. The fluorescent antibody signal was superimposed on a false colour transformation of the *Plexin-A2 in situ *signal ((e,f) red). This shows that when superimposed (e) there is little overlap of the two colours, confirming the efficacy of the *Plexin-A2 *knockdown. Bar = 100 μm. Click here for file

Additional file 7 *Egr2/Krox-20 *expressing BC cells persist in *Npn-2 *mutant mice. *In situ *hybridisation for *Egr2 *on transverse cryosections (30 μm) obtained from E11.5 mouse embryos at hindlimb level. The results show no obvious difference in *Egr2 *expression in BC cells located at the DREZ or MEP in **(a) **wild-type, **(b) **heterozygous or **(c) ***Npn-2 *null mice. Bar = 100 μm. Click here for file

Additional file 8 Quantification of the number of ectopic motor neurons in chick embryos electroporated in the ventral neural tube with EGFP-shRNAs. Table providing a quantitative analysis of the occurrence of ectopic motor neurons after electroporation of chick embryos in the ventral neural tube with *Npn-2*, *Plexin-A2, MICAL3 *EGFP-shRNA or empty vector. Embryos were electroporated and processed as described in the legend to Figure 2. Click here for file

## References

[B1] Ridley AJ, Schwartz MA, Burridge K, Firtel RA, Ginsberg MH, Borisy G, Parsons JT, Horwitz AR (2003). Cell migration: integrating signals from front to back. Science.

[B2] Edmondson JC, Hatten ME (1987). Glial-guided granule neuron migration *in vitro*: a high-resolution time-lapse video microscopic study. J Neurosci.

[B3] Lambert de Rouvroit C, Goffinet AM (2001). Neuronal migration. Mech Dev.

[B4] Ayala R, Shu T, Tsai LH (2007). Trekking across the brain: the journey of neuronal migration. Cell.

[B5] Jessell TM (2000). Neuronal specification in the spinal cord: inductive signals and transcriptional codes. Nat Rev Genet.

[B6] Arber S, Ladle DR, Lin JH, Frank E, Jessell TM (2000). ETS gene Er81 controls the formation of functional connections between group Ia sensory afferents and motor neurons. Cell.

[B7] Kania A, Jessell TM (2003). Topographic motor projections in the limb imposed by LIM homeodomain protein regulation of ephrin-A:EphA interactions. Neuron.

[B8] Dillon AK, Fujita SC, Matise MP, Jarjour AA, Kennedy TE, Kollmus H, Arnold HH, Weiner JA, Sanes JR, Kaprielian Z (2005). Molecular control of spinal accessory motor neuron/axon development in the mouse spinal cord. J Neurosci.

[B9] Lieberam I, Agalliu D, Nagasawa T, Ericson J, Jessell TM (2005). A Cxcl12-CXCR4 chemokine signaling pathway defines the initial trajectory of mammalian motor axons. Neuron.

[B10] Price SR, De Marco Garcia NV, Ranscht B, Jessell TM (2002). Regulation of motor neuron pool sorting by differential expression of type II cadherins. Cell.

[B11] Feldner J, Becker T, Goishi K, Schweitzer J, Lee P, Schachner M, Klagsbrun M, Becker CG (2005). Neuropilin-1a is involved in trunk motor axon outgrowth in embryonic zebrafish. Dev Dyn.

[B12] Mohamed AM, Chin-Sang ID (2006). Characterization of loss-of-function and gain-of-function Eph receptor tyrosine kinase signaling in *C. elegans *axon targeting and cell migration. Dev Biol.

[B13] Huber AB, Kania A, Tran TS, Gu C, De Marco Garcia N, Lieberam I, Johnson D, Jessell TM, Ginty DD, Kolodkin AL (2005). Distinct roles for secreted semaphorin signaling in spinal motor axon guidance. Neuron.

[B14] Cohen S, Funkelstein L, Livet J, Rougon G, Henderson CE, Castellani V, Mann F (2005). A semaphorin code defines subpopulations of spinal motor neurons during mouse development. Eur J Neurosci.

[B15] Mauti O, Sadhu R, Gemayel J, Gesemann M, Stoeckli ET (2006). Expression patterns of plexins and neuropilins are consistent with cooperative and separate functions during neural development. BMC Dev Biol.

[B16] Marin O, Yaron A, Bagri A, Tessier-Lavigne M, Rubenstein JL (2001). Sorting of striatal and cortical interneurons regulated by semaphorin-neuropilin interactions. Science.

[B17] Kerjan G, Dolan J, Haumaitre C, Schneider-Maunoury S, Fujisawa H, Mitchell KJ, Chedotal A (2005). The transmembrane semaphorin Sema6A controls cerebellar granule cell migration. Nat Neurosci.

[B18] Vermeren M, Maro GS, Bron R, McGonnell IM, Charnay P, Topilko P, Cohen J (2003). Integrity of developing spinal motor columns is regulated by neural crest derivatives at motor exit points. Neuron.

[B19] Bron R, Eickholt BJ, Vermeren M, Fragale N, Cohen J (2004). Functional knockdown of Neuropilin-1 in the developing chick nervous system by siRNA hairpins phenocopies genetic ablation in the mouse. Dev Dyn.

[B20] McLennan R, Kulesa PM (2006). *In vivo *analysis reveals a critical role for neuropilin-1 in cranial neural crest cell migration in chick. Dev Biol.

[B21] Eickholt BJ, Mackenzie SL, Graham A, Walsh FS, Doherty P (1999). Evidence for collapsin-1 functioning in the control of neural crest migration in both trunk and hindbrain regions. Development.

[B22] Gammill LS, Gonzalez C, Gu C, Bronner-Fraser M (2005). Guidance of trunk neural crest migration requires neuropilin 2/semaphorin 3F signaling. Development.

[B23] Takahashi T, Fournier A, Nakamura F, Wang LH, Murakami Y, Kalb RG, Fujisawa H, Strittmatter SM (1999). Plexin-neuropilin-1 complexes form functional semaphorin-3A receptors. Cell.

[B24] Tran TS, Kolodkin AL, Bharadwaj R (2007). Semaphorin regulation of cellular morphology. Annu Rev Cell Dev Biol.

[B25] Giger RJ, Cloutier JF, Sahay A, Prinjha RK, Levengood DV, Moore SE, Pickering S, Simmons D, Rastan S, Walsh FS (2000). Neuropilin-2 is required *in vivo *for selective axon guidance responses to secreted semaphorins. Neuron.

[B26] Chen H, Bagri A, Zupicich JA, Zou Y, Stoeckli E, Pleasure SJ, Lowenstein DH, Skarnes WC, Chedotal A, Tessier-Lavigne M (2000). Neuropilin-2 regulates the development of selective cranial and sensory nerves and hippocampal mossy fiber projections. Neuron.

[B27] Falk J, Bechara A, Fiore R, Nawabi H, Zhou H, Hoyo-Becerra C, Bozon M, Rougon G, Grumet M, Puschel AW (2005). Dual functional activity of semaphorin 3B is required for positioning the anterior commissure. Neuron.

[B28] Gammill LS, Gonzalez C, Bronner-Fraser M (2007). Neuropilin 2/semaphorin 3F signaling is essential for cranial neural crest migration and trigeminal ganglion condensation. Dev Neurobiol.

[B29] Maro GS, Vermeren M, Voiculescu O, Melton L, Cohen J, Charnay P, Topilko P (2004). Neural crest boundary cap cells constitute a source of neuronal and glial cells of the PNS. Nat Neurosci.

[B30] Taniguchi M, Masuda T, Fukaya M, Kataoka H, Mishina M, Yaginuma H, Watanabe M, Shimizu T (2005). Identification and characterization of a novel member of murine semaphorin family. Genes Cells.

[B31] Suto F, Ito K, Uemura M, Shimizu M, Shinkawa Y, Sanbo M, Shinoda T, Tsuboi M, Takashima S, Yagi T, Fujisawa H (2005). Plexin-a4 mediates axon-repulsive activities of both secreted and transmembrane semaphorins and plays roles in nerve fiber guidance. J Neurosci.

[B32] Suto F, Tsuboi M, Kamiya H, Mizuno H, Kiyama Y, Komai S, Shimizu M, Sanbo M, Yagi T, Hiromi Y (2007). Interactions between Plexin-A2, Plexin-A4, and Semaphorin 6A control lamina-restricted projection of hippocampal mossy fibers. Neuron.

[B33] Mitchell KJ, Pinson KI, Kelly OG, Brennan J, Zupicich J, Scherz P, Leighton PA, Goodrich LV, Lu X, Avery BJ (2001). Functional analysis of secreted and transmembrane proteins critical to mouse development. Nat Genet.

[B34] Terman JR, Mao T, Pasterkamp RJ, Yu HH, Kolodkin AL (2002). MICALs, a family of conserved flavoprotein oxidoreductases, function in plexin-mediated axonal repulsion. Cell.

[B35] Pasterkamp RJ, Dai HN, Terman JR, Wahlin KJ, Kim B, Bregman BS, Popovich PG, Kolodkin AL (2006). MICAL flavoprotein monooxygenases: expression during neural development and following spinal cord injuries in the rat. Mol Cell Neurosci.

[B36] Boardman PE, Sanz-Ezquerro J, Overton IM, Burt DW, Bosch E, Fong WT, Tickle C, Brown WR, Wilson SA, Hubbard SJ (2002). A comprehensive collection of chicken cDNAs. Curr Biol.

[B37] Fache MP, Moussif A, Fernandes F, Giraud P, Garrido JJ, Dargent B (2004). Endocytotic elimination and domain-selective tethering constitute a potential mechanism of protein segregation at the axonal initial segment. J Cell Biol.

[B38] Jacobson C, Schnapp B, Banker GA (2006). A change in the selective translocation of the Kinesin-1 motor domain marks the initial specification of the axon. Neuron.

[B39] Polleux F, Morrow T, Ghosh A (2000). Semaphorin 3A is a chemoattractant for cortical apical dendrites. Nature.

[B40] Swartz M, Eberhart J, Mastick GS, Krull CE (2001). Sparking new frontiers: using *in vivo *electroporation for genetic manipulations. Dev Biol.

[B41] Reynolds A, Leake D, Boese Q, Scaringe S, Marshall WS, Khvorova A (2004). Rational siRNA design for RNA interference. Nat Biotechnol.

[B42] Ding Y, Chan CY, Lawrence CE (2004). Sfold web server for statistical folding and rational design of nucleic acids. Nucleic Acids Res.

[B43] Alvarez VA, Ridenour DA, Sabatini BL (2006). Retraction of synapses and dendritic spines induced by off-target effects of RNA interference. J Neurosci.

[B44] Birmingham A, Anderson EM, Reynolds A, Ilsley-Tyree D, Leake D, Fedorov Y, Baskerville S, Maksimova E, Robinson K, Karpilow J (2006). 3' UTR seed matches, but not overall identity, are associated with RNAi off-targets. Nat Methods.

[B45] Grimm D, Streetz KL, Jopling CL, Storm TA, Pandey K, Davis CR, Marion P, Salazar F, Kay MA (2006). Fatality in mice due to oversaturation of cellular microRNA/short hairpin RNA pathways. Nature.

[B46] Toyofuku T, Zhang H, Kumanogoh A, Takegahara N, Suto F, Kamei J, Aoki K, Yabuki M, Hori M, Fujisawa H, Kikutani H (2004). Dual roles of Sema6D in cardiac morphogenesis through region-specific association of its receptor, Plexin-A1, with off-tack and vascular endothelial growth factor receptor type 2. Genes Dev.

[B47] Cheng HJ, Bagri A, Yaron A, Stein E, Pleasure SJ, Tessier-Lavigne M (2001). Plexin-A3 mediates semaphorin signaling and regulates the development of hippocampal axonal projections. Neuron.

[B48] Yaron A, Huang PH, Cheng HJ, Tessier-Lavigne M (2005). Differential requirement for plexin-a3 and -a4 in mediating responses of sensory and sympathetic neurons to distinct class 3 semaphorins. Neuron.

[B49] Yoshida Y, Han B, Mendelsohn M, Jessell TM (2006). PlexinA1 signaling directs the segregation of proprioceptive sensory axons in the developing spinal cord. Neuron.

[B50] Kolk SM, Pasterkamp RJ (2007). MICAL flavoprotein monooxygenases: structure, function and role in semaphorin signaling. Adv Exp Med Biol.

[B51] Goshima Y, Nakamura F, Strittmatter P, Strittmatter SM (1995). Collapsin-induced growth cone collapse mediated by an intracellular protein related to UNC-33. Nature.

[B52] Gu Y, Ihara Y (2000). Evidence that collapsin response mediator protein-2 is involved in the dynamics of microtubules. J Biol Chem.

[B53] Wang LH, Strittmatter SM (1996). A family of rat CRMP genes is differentially expressed in the nervous system. J Neurosci.

[B54] Bretin S, Reibel S, Charrier E, Maus-Moatti M, Auvergnon N, Thevenoux A, Glowinski J, Rogemond V, Premont J, Honnorat J, Gauchy C (2005). Differential expression of CRMP1, CRMP2A, CRMP2B, and CRMP5 in axons or dendrites of distinct neurons in the mouse brain. J Comp Neurol.

[B55] Tanaka T, Serneo FF, Higgins C, Gambello MJ, Wynshaw-Boris A, Gleeson JG (2004). Lis1 and doublecortin function with dynein to mediate coupling of the nucleus to the centrosome in neuronal migration. J Cell Biol.

[B56] Solecki DJ, Model L, Gaetz J, Kapoor TM, Hatten ME (2004). Par6alpha signaling controls glial-guided neuronal migration. Nat Neurosci.

[B57] Fischer J, Weide T, Barnekow A (2005). The MICAL proteins and rab1: a possible link to the cytoskeleton?. Biochem Biophys Res Commun.

[B58] Yazdani U, Terman JR (2006). The semaphorins. Genome Biol.

[B59] Gallo G (2006). RhoA-kinase coordinates F-actin organization and myosin II activity during semaphorin-3A-induced axon retraction. J Cell Sci.

[B60] Lee A, Treisman JE (2004). Excessive Myosin activity in mbs mutants causes photoreceptor movement out of the *Drosophila *eye disc epithelium. Mol Biol Cell.

[B61] Myers KA, Tint I, Nadar CV, He Y, Black MM, Baas PW (2006). Antagonistic forces generated by cytoplasmic dynein and myosin-II during growth cone turning and axonal retraction. Traffic.

[B62] Fan SS, Ready DF (1997). Glued participates in distinct microtubule-based activities in *Drosophila *eye development. Development.

[B63] Whited JL, Cassell A, Brouillette M, Garrity PA (2004). Dynactin is required to maintain nuclear position within postmitotic *Drosophila *photoreceptor neurons. Development.

[B64] Tsai JW, Bremner KH, Vallee RB (2007). Dual subcellular roles for LIS1 and dynein in radial neuronal migration in live brain tissue. Nat Neurosci.

[B65] Golding JP, Cohen J (1997). Border controls at the mammalian spinal cord: late-surviving neural crest boundary cap cells at dorsal root entry sites may regulate sensory afferent ingrowth and entry zone morphogenesis. Mol Cell Neurosci.

[B66] Cariboni A, Hickok J, Rakic S, Andrews W, Maggi R, Tischkau S, Parnavelas JG (2007). Neuropilins and their ligands are important in the migration of gonadotropin-releasing hormone neurons. J Neurosci.

[B67] Leighton PA, Mitchell KJ, Goodrich LV, Lu X, Pinson K, Scherz P, Skarnes WC, Tessier-Lavigne M (2001). Defining brain wiring patterns and mechanisms through gene trapping in mice. Nature.

[B68] Yaneza M, Gilthorpe JD, Lumsden A, Tucker AS (2002). No evidence for ventrally migrating neural tube cells from the mid- and hindbrain. Dev Dyn.

[B69] Sui G, Soohoo C, Affar el B, Gay F, Shi Y, Forrester WC, Shi Y (2002). A DNA vector-based RNAi technology to suppress gene expression in mammalian cells. Proc Natl Acad Sci USA.

[B70] SFOLD. http://sfold.wadsworth.org.

[B71] Ambion pSilencer Insert Designer. http://www.ambion.com/techlib/misc/psilencer_converter.html.

[B72] Myat A, Henrique D, Ish-Horowicz D, Lewis J (1996). A chick homologue of Serrate and its relationship with Notch and Delta homologues during central neurogenesis. Dev Biol.

[B73] Strahle U, Blader P, Adam J, Ingham PW (1994). A simple and efficient procedure for non-isotopic *in situ *hybridization to sectioned material. Trends Genet.

[B74] Osborne NJ, Begbie J, Chilton JK, Schmidt H, Eickholt BJ (2005). Semaphorin/neuropilin signaling influences the positioning of migratory neural crest cells within the hindbrain region of the chick. Dev Dyn.

