# Supplementary figures and images for: Boundary cap cells constrain spinal motor neuron somal migration at motor exit points by a semaphorin-plexin mechanism
Source: Neural Dev. 2007 Oct 30;2:21. doi: 10.1186/1749-8104-2-21 (PMC2131750; doi:10.1186/1749-8104-2-21)

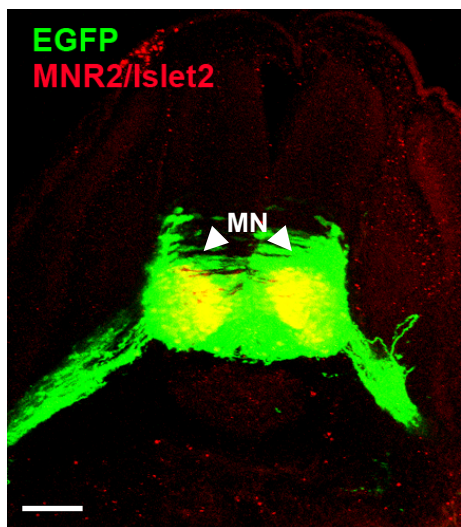

Ventral

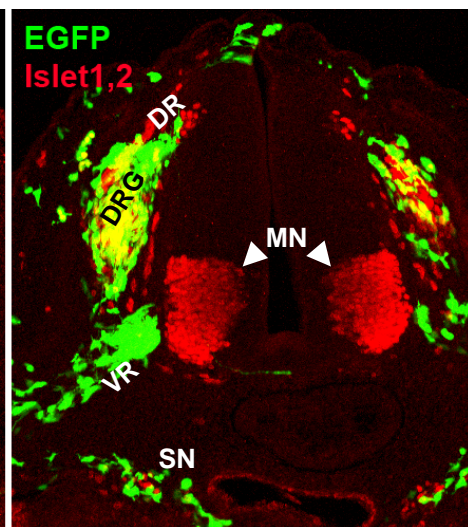

Dorsal (Neural Crest)

Supplement: Additional file 1 — Targeted delivery of EGFP expression plasmids by selective electroporation towards either the ventral neural tube or the neural crest. Confocal micrographs of transverse vibratome sections (75 μm). (a) An example of a HH23 chick embryo electroporated in the ventral neural tube with the pCA-EGFPm5-mU6 plasmid, as detailed in the Materials and methods. Motor neurons are labelled with a mixture of anti- MNR2 and Islet-2 antibodies (red). Note that virtually all islet-2/MNR2 positive motor neurons (MN) in this section appear yellow, indicating co-expression of EGFP. No EGFP expression is seen outside the neural tube. (b) The plasmid was targeted towards the dorsal neural tube. The section shown was taken several somites away from the electroporation site and shows EGFP expression in neural crest derivates only (dorsal root ganglion (DRG), sympathetic ganglia (Sn) and glia in ventral (VR) and dorsal (DR) spinal nerve roots). The anti-Islet-1,2 antibody (red) labels motor neurons, DRG neurons, sympathetic neurons and dorsal horn interneurons. Note the complete absence of EGFP expression within the spinal cord. Bar = 100 μm. [file 1749-8104-2-21-S1.pdf]

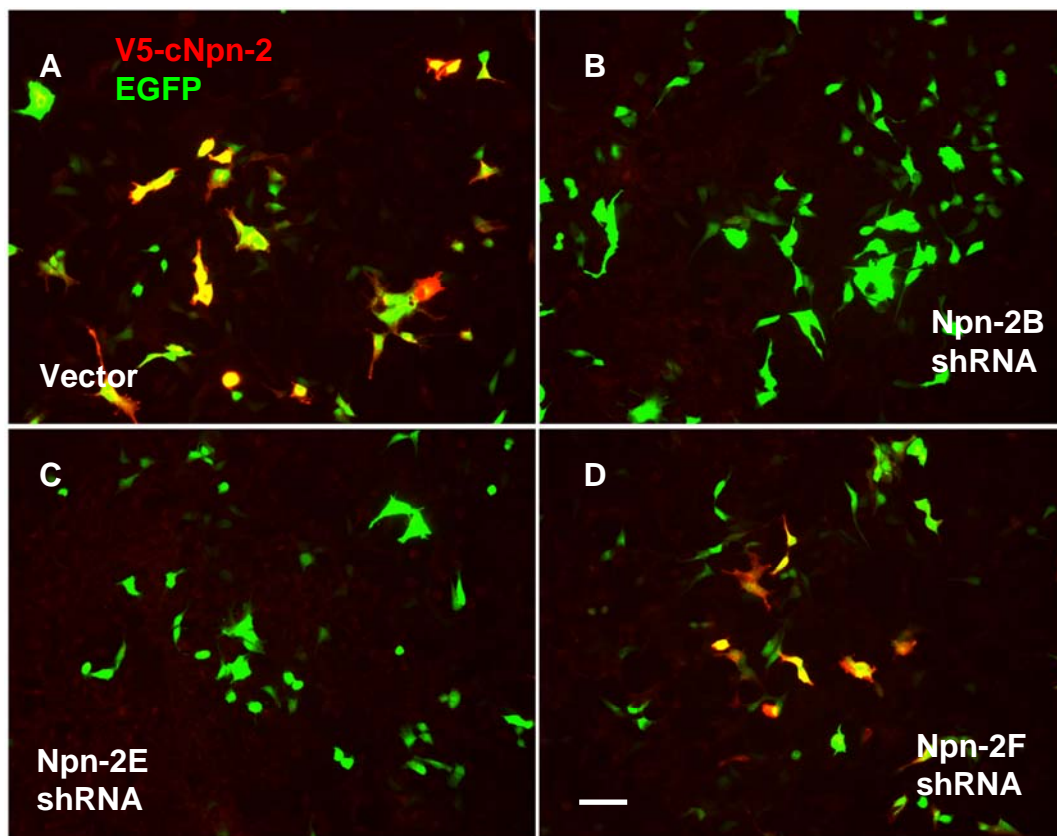

Supplement: Additional file 2 — Npn-2 B and Npn-2 E shRNA reduce expression of V5-tagged cNpn-2 in HEK cells. Vectors co-expressing EGFP and shRNA targeted at Npn-2 were co-transfected with pcDNA3.1-V5-cNpn-2 [19] at a ratio of 5:1. At 2 days post-transfection, cells were fixed and stained with V5 antibodies. The data show efficient knockdown of V5-tagged Npn-2 with Npn-2 B and Npn-2 E shRNA. By contrast, the Npn-2 F shRNA construct is ineffective. Bar = 25 μm. [file 1749-8104-2-21-S2.pdf]

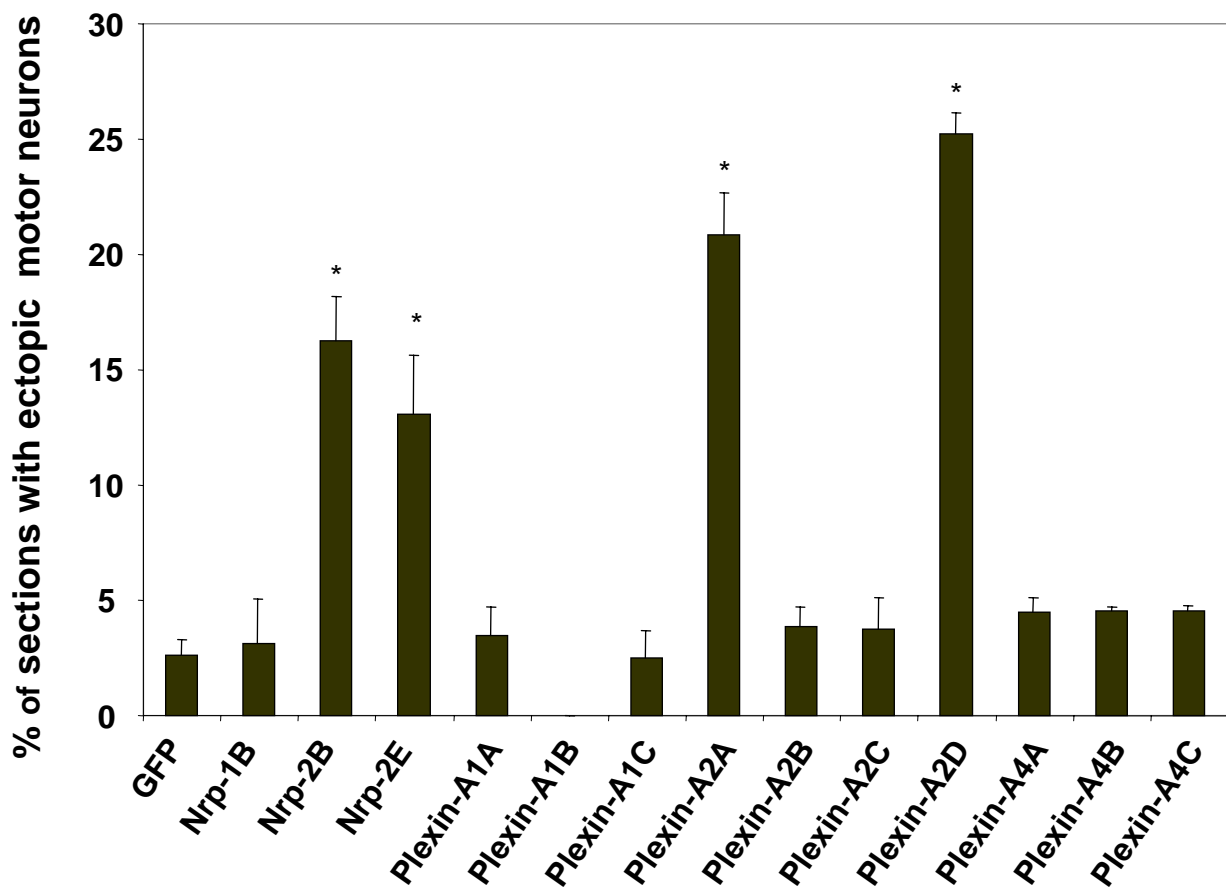

Supplement: Additional file 3 — Screen of shRNA vectors targeting Npn or Plexin-A receptors in the chick for the ability to induce ectopic motor neuron migration. The prevalence of ectopic motor neurons after ventral electroporation of shRNA vectors targeting plexin-A and neuropilin receptors in the chick was assessed. In a first round of analysis, three different vectors were tested for each Plexin-A target along with validated npn-1 and Npn-2 shRNA vectors. After obtaining positive results for Npn-2B and Plexin-A2A shRNA, a second batch of triplicate vectors targeting Npn-2 and Plexin-A2 was tested. Of these vectors Npn-2 E and Plexin-A2D shRNA also induced ectopic motor neurons, whilst Npn-2 C, -2D, -2F and Plexin-A2E and -A2F constructs were ineffective. ***P < 0.001; two-tailed t-test. [file 1749-8104-2-21-S3.pdf]

plexin-A1A  
shRNA

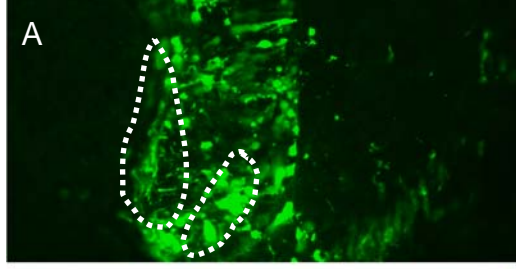

plexin-A2A  
shRNA

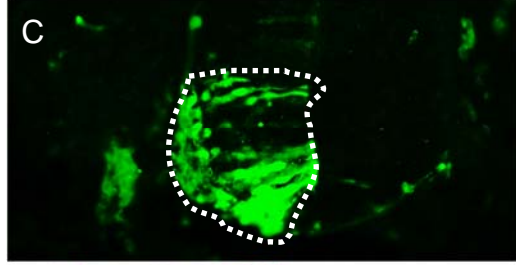

plexin-A4C  
shRNA

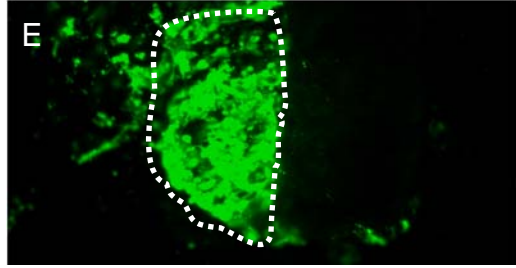

plexin-A1

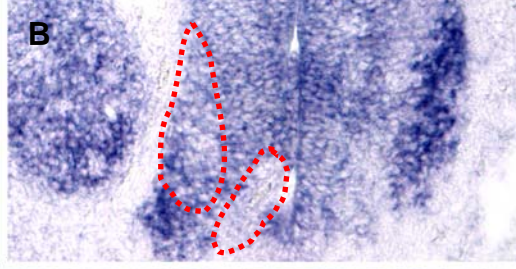

plexin-A2

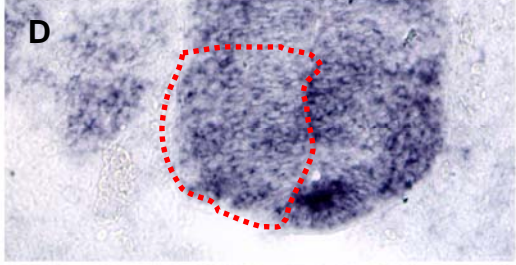

plexin-A4

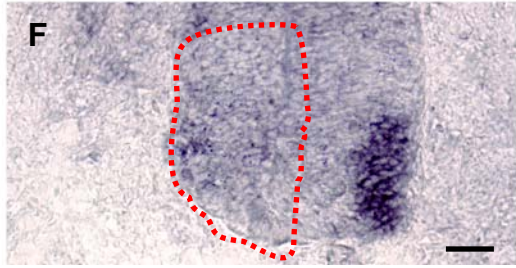

Supplement: Additional file 5 — Confirmation of knockdown of Plexin mRNAs by ventral targeted shRNA in the chick spinal cord. Embryos were electroporated at E2 (HH stage 13–15) in the ventral neural tube with shRNA vectors targeting (a,b) Plexin-A1, (c,d) Plexin-A2 or (e,f) Plexin-A4. The effect of each shRNA on expression was assessed after 48–54 hours (HH 23–24) by in situ hybridisation on transverse cryosections of hindlimb region spinal cord (20 μm) (b,d,f). Bar = 100 μm. The data show a clear reduction of the in situ signals of the targeted genes in the electroporated areas (enclosed by dashed lines), compared to corresponding regions on the un-electroporated, contralateral side. [file 1749-8104-2-21-S5.pdf]

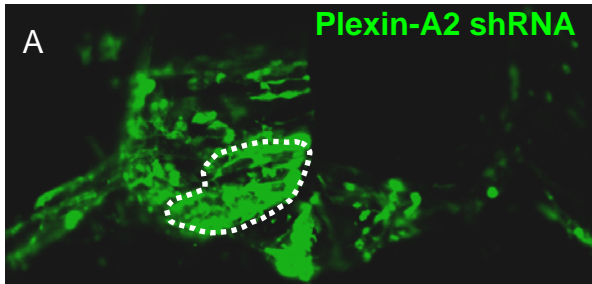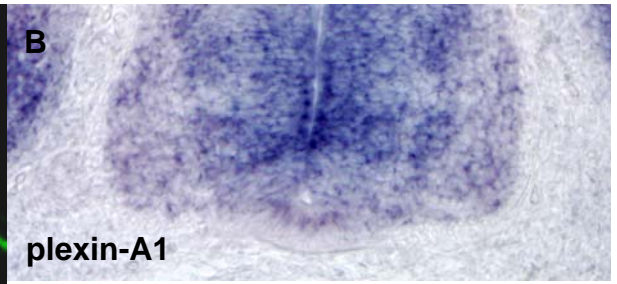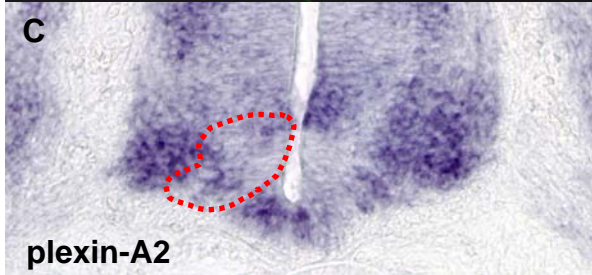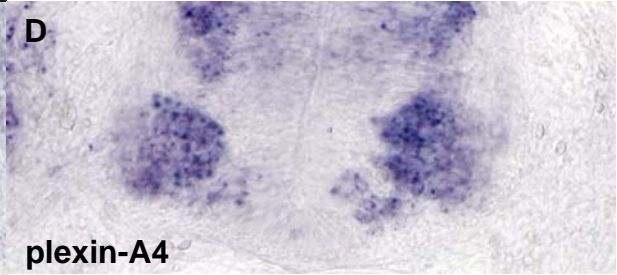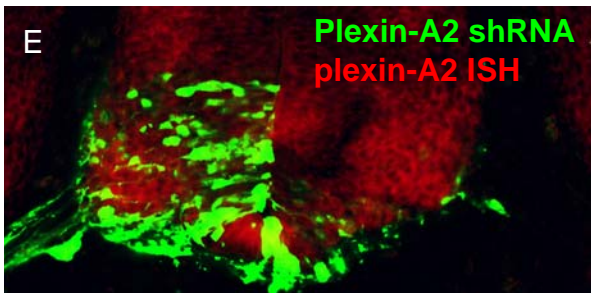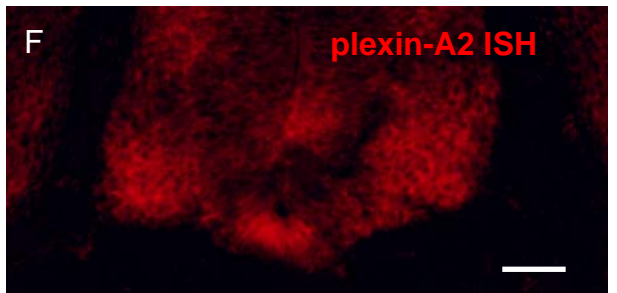

Supplement: Additional file 6 — Specificity of knockdown by Plexin-A2 shRNA. An embryo was electroporated at E2 (HH stage 13–15) in the ventral neural tube with an shRNA vector targeting Plexin-A2. The effect at HH stage 23 of shRNA electroporation (a) on expression of (b) Plexin-A1, (c) -A2 and (d) -A4 was assessed on adjacent transverse cryosections of hindlimb region spinal cord (30 μm). Plexin-A2 shRNA was effective in reducing Plexin-A2 mRNA (c) (marked knockdown evident in regions enclosed by dotted lines in (a,c)) but was without effect on expression of Plexin-A1 (b) and Plexin-A4 (d). (e) To more precisely map the region of Plexin-A2 shRNA expression onto the Plexin-A2 gene expression pattern, EGFP was visualised by GFP antibody labelling (green) post hybridisation on a section from the same embryo as above. The fluorescent antibody signal was superimposed on a false colour transformation of the Plexin-A2 in situ signal ((e,f) red). This shows that when superimposed (e) there is little overlap of the two colours, confirming the efficacy of the Plexin-A2 knockdown. Bar = 100 μm. [file 1749-8104-2-21-S6.pdf]

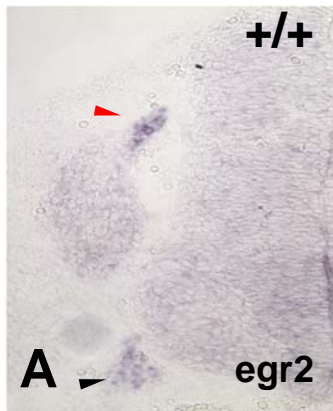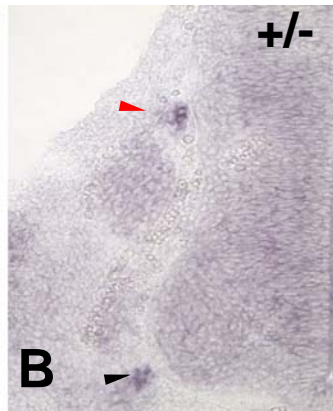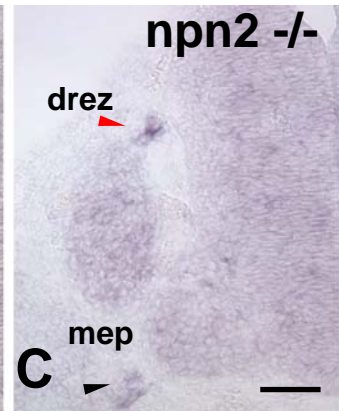

Supplement: Additional file 7 — Egr2/Krox-20 expressing BC cells persist in Npn-2 mutant mice. In situ hybridisation for Egr2 on transverse cryosections (30 μm) obtained from E11.5 mouse embryos at hindlimb level. The results show no obvious difference in Egr2 expression in BC cells located at the DREZ or MEP in (a) wild-type, (b) heterozygous or (c) Npn-2 null mice. Bar = 100 μm. [file 1749-8104-2-21-S7.pdf]
